# Engineered macrophages with IL-10–TLR9 signal switch receptors for reprogramming tumor microenvironment and enhancing antitumor immunity

**DOI:** 10.1038/s12276-026-01800-5

**Published:** 2026-08-06

**Authors:** Sijie Wang, Owais Ahmad, Kexin Shui, Huifang Su, Rui Wang, Qing Wang, Wenlong Zhang, Qing Zhang, Hongqian Guo, Pingping Shen

**Affiliations:** 1 Department of Urology, Nanjing Drum Tower Hospital, Affiliated Hospital of Medical School, Nanjing University, Nanjing, Jiangsu China; 2 The State Key Laboratory of Pharmaceutical Biotechnology, School of Life Sciences, Nanjing University, Nanjing, Jiangsu China; 3 Macera Therapeutics, Nanjing, Jiangsu China

**Keywords:** Synthetic biology, Breast cancer, Genetic engineering, Immunotherapy

## Abstract

Chimeric antigen receptor (CAR) T cell immunotherapy has achieved remarkable success in hematologic malignancies, prompting the exploration of CAR strategies in solid tumors. Here, we developed a CAR-like signal-switching receptor-macrophage (SR CAR-M) that recognizes IL-10, a major immunosuppressive cytokine enriched in solid tumors, and converts this inhibitory signal into a pro-inflammatory activation through the Toll-like receptor 9 (TLR9) intracellular signaling pathway. SR CAR-Ms effectively blocked IL-10-mediated STAT3 phosphorylation while activating TLR9 downstream signaling, including nuclear translocation of nuclear factor-κB and upregulation of IRF1, thereby adopting an M1-like phenotype with enhanced phagocytic capacity and tumor cytotoxicity. Domain-deletion controls demonstrated that both IL-10 binding and TLR9 signaling are indispensable. In addition, SR CAR-Ms promoted dendritic cell maturation, enhanced T cell proliferation and effector function, and prevented T cell exhaustion. In an orthotopic 4T1 breast cancer model, infused SR CAR-Ms selectively accumulated in tumors, depleted local IL-10 while inducing inflammatory cytokine production, suppressed tumor growth, and prolonged survival without systemic toxicity. The signal-switching mechanism was validated in primary bone marrow-derived macrophages and human monocyte-derived macrophages, supporting the translational applicability. Further enhancement was achieved by engineering dual-function SRPα CAR-Ms secreting anti-PD-L1 antibodies, which outperformed either SR CAR-M or anti-PD-L1 monotherapy. The SR CAR-M platform transforms an immunosuppressive cytokine into a location-specific activation trigger, simultaneously depleting the inhibitory signal and remodeling the tumor microenvironment. This signal-switching paradigm offers a versatile approach for treating solid malignancies that are resistant to conventional immunotherapies.

**Figure:**
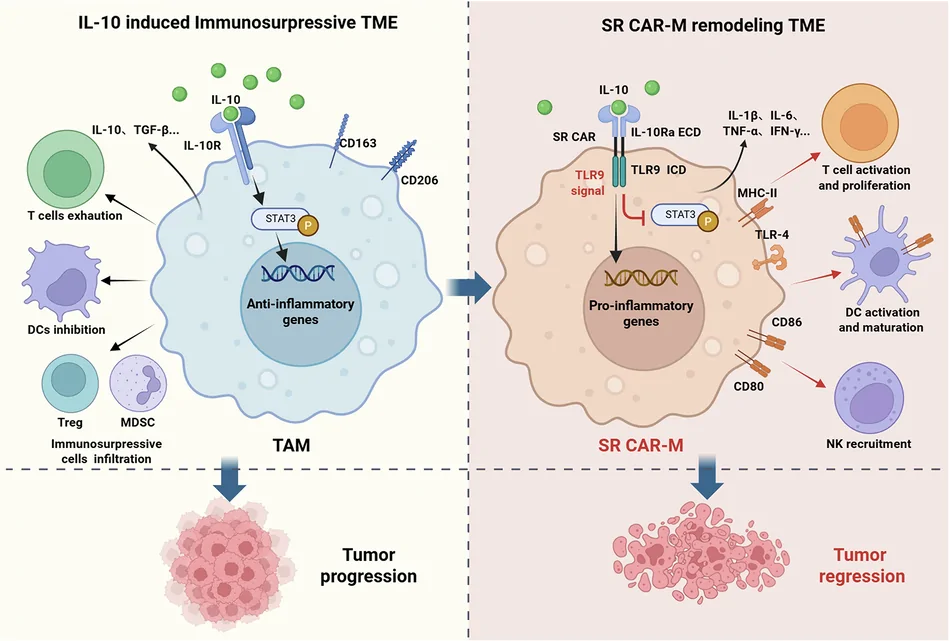

## Transforming immunosuppressive signals into tumor fighting power

Cancer immunotherapy, particularly CAR-T cell therapy, has transformed the treatment of blood cancers but struggles with solid tumors owing to the immunosuppressive nature of the tumor microenvironment (TME). This study explores CAR-macrophage (CAR-M) therapy as an alternative, leveraging macrophages’ natural tumor-homing and immune-modulating abilities. Researchers developed a chimeric switch receptor (SR CAR-M) that converts the immunosuppressive cytokine IL-10 into a pro-inflammatory signal, enhancing macrophage antitumor activity. In vitro and in vivo experiments showed SR CAR-Ms maintained an M1-like phenotype, resisted reprogramming, and improved phagocytosis and cytotoxicity against tumor cells. Importantly, SR CAR-Ms infiltrated tumors, suppressed growth, and prolonged survival in mice without systemic toxicity. This approach reprograms the TME, enhancing antitumor immunity and offering a promising strategy for solid tumor treatment. Future directions include optimizing tumor-specific trafficking and exploring other immunosuppressive factors for similar therapeutic strategies. DC, dendritic cell; IFN, interferon; MDSC, myeloid-derived suppressor cell; MHC, major histocompatibility complex; NK, natural killer; SR CAR-M, switch receptor chimeric antigen receptor-macrophage; TAM, tumor-associated macrophage; TGF-β, transforming growth factor-beta; TLR, Toll-like receptor; TNF, tumor necrosis factor; T_reg_, regulatory T.

This summary was initially drafted using artificial intelligence, then revised and fact-checked by the author.

## Introduction

Cancer immunotherapy has revolutionized oncology, with chimeric antigen receptor T (CAR-T) cell therapy achieving unprecedented success in the treatment of hematological malignancies^1,2^. Despite remarkable clinical outcomes, the application of CAR-T therapy to solid tumors is profoundly limited by multiple formidable barriers^3,4^. The immunosuppressive tumor microenvironment (TME), inadequate T cell infiltration, antigen heterogeneity, and limited persistence of infused cells collectively impede the therapeutic efficacy of solid tumors^5^. As of 2025, despite extensive clinical investigations with more than 100 registered trials, CAR-T therapies have failed to achieve consistent curative responses in solid tumors, underscoring the urgent need for alternative cellular immunotherapy platforms^6,7^.

CAR-macrophage (CAR-M) therapy has emerged as a promising alternative approach that leverages the unique biological properties of macrophages to overcome the limitations encountered by CAR-T cells^8,9^. Unlike T lymphocytes, macrophages possess inherent tumor-homing capabilities, robust phagocytic capacity, antigen-presenting functions, and the ability to remodel the immunosuppressive TME^10^. The first-in-human phase I clinical trial of CT-0508, an autologous HER2-targeted CAR-M product, demonstrated favorable safety profiles with no dose-limiting toxicities, grade ≥3 cytokine release syndrome, or neurotoxicity, establishing its clinical feasibility^11^. Notably, 44% of patients with HER2 3+ tumors achieved stable disease, and correlative analyses confirmed CAR-M trafficking to tumors, TME remodeling, and CD8^+^ T cell expansion. Currently, six CAR-M clinical trials are underway, targeting various solid tumor antigens, including HER2, mesothelin, and GPC3, reflecting the growing recognition of the potential of this platform^10–12^.

However, tumor-associated macrophages (TAMs) typically adopt an immunosuppressive M2-like phenotype that actively promotes tumor progression^13,14^. Among the myriad factors orchestrating this immunosuppression, IL-10 emerges as a particularly potent mediator with pleiotropic effects on multiple immune cell populations^15,16^. IL-10 is abundantly secreted by diverse tumor types and functions as a master regulator of immune evasion by inhibiting dendritic cell (DC) maturation, suppressing effector T cell function, promoting regulatory T cell differentiation, and reinforcing M2 macrophage polarization^17^. Elevated IL-10 expression correlates with poor prognosis in multiple cancer types, and immunosuppressive TAMs expressing IL-10 confer therapeutic vulnerability in bladder cancer and other malignancies^18^. Recent studies have demonstrated that IL-10 dampens antitumor immunity via PD-L1 induction and facilitates liver metastasis, whereas myeloid cell-derived IL-10 mediates T cell dysfunction in glioblastoma^18,19^. The dual challenge posed by IL-10, as both a critical immunosuppressive signal and a ubiquitous component of the TME, necessitates innovative therapeutic strategies^20^.

Signal-switching technology represents a paradigm-shifting approach to cellular immunotherapy, enabling engineered cells to convert hostile microenvironmental cues into therapeutic activation signals^21,22^. The concept of inverted cytokine receptors was pioneered through the development of chimeric receptors that fuse immunosuppressive cytokine-binding domains to activating signaling moieties^23,24^. Mohammed et al.^23^ demonstrated that linking the IL-4 receptor extracellular domain to the IL-7 receptor endodomain inverted immunosuppression into T cell proliferation and activation. This principle has been successfully extended to multiple immunoinhibitory factors, with chimeric switch receptors targeting PD-1, TIGIT, transforming growth factor-beta (TGF-β), and other TME-enriched molecules, enhancing engineered T cell efficacy^22,25,26^. Recent advances include the development of a chimeric cytokine receptor (G6/7R) that captures IL-6 while delivering IL-7 signaling, simultaneously neutralizing pro-inflammatory cytokines and enhancing CAR-T cell persistence^27^. Notably, signal-switching CAR-Ms that recognize tumor necrosis factor (TNF) and activate IL-4 signaling have demonstrated therapeutic efficacy in kidney injury models, validating this approach in myeloid cells^28^.

Despite these advances, no published studies have successfully engineered macrophages to exploit IL-10, arguably the most prevalent and potent immunosuppressive cytokine in solid tumors, as a location-specific activation trigger. Converting IL-10 from a liability to a therapeutic asset would address multiple challenges simultaneously: depleting local IL-10, activating macrophages selectively within tumors, and maintaining M1 phenotype stability despite persistent immunosuppressive pressure. In this study, we developed a CAR-like switch receptor construct (SR CAR) that fuses the IL-10 receptor exodomain to the TLR9 receptor endodomain, creating SR CAR-M that converts IL-10 signals into pro-inflammatory TLR9 pathway activation. We demonstrated that SR CAR-M cells exhibit an M1-like phenotype, enhanced phagocytosis and cytotoxicity, resistance to microenvironmental reprogramming, promotion of DC maturation and T cell activation, selective tumor infiltration, and potent antitumor efficacy in an orthotopic 4T1 breast cancer model. Furthermore, we engineered dual-functional SR CAR-M that secretes anti-PD-L1 antibodies (SRPα CAR-M), achieving superior tumor control through synchronized microenvironment remodeling and checkpoint blockade. Our findings establish signal switching as a powerful strategy for engineering tumor-selective macrophage immunotherapy, transforming the most abundant immunosuppressive signal in solid tumors into a therapeutic activation mechanism.

## Materials and methods

### Cell lines and cell culture

The murine cancer cell lines B16F10 (melanoma), LLC (lung), MB49 (bladder), 4T1 (breast), and Raw264.7 (murine macrophage), the human breast cancer cell line MDA-MB-231, and the human embryonic kidney cell line HEK293T were procured from the China Center for Type Culture Collection (Wuhan, China). Immortalized bone marrow-derived macrophages (iBMDMs) were obtained from the Shao Feng Laboratory (National Institute of Biological Sciences, Beijing, China). Cancer cell lines were maintained in RPMI 1640 medium, whereas Raw264.7 cells and iBMDMs were cultured in Dulbecco’s modified Eagle medium (DMEM), both supplemented with 10% fetal bovine serum (FBS; Gibco), 2 mmol/l L-glutamine, 100 IU/ml penicillin, and 100 IU/ml streptomycin. All cultures were incubated at 37 °C in a humidified atmosphere with 5% CO_2_. Cell lines were authenticated via short tandem repeat DNA fingerprinting at the Key Laboratory of Pharmaceutical Biotechnology and the Comprehensive Cancer Center of Nanjing University (Nanjing, China) and confirmed to be free of mycoplasma contamination using a PCR-based detection kit. To ensure consistency, no cell lines were passaged beyond 3 months post-resuscitation.

### Immunofluorescence

To assess nuclear factor (NF)-κB activation, mouse macrophages, primary BMDMs, or human monocyte-derived macrophages were seeded onto cell-climbing tablets in 12-well culture plates. Cells were fixed with 4% paraformaldehyde for 30 min, washed twice with phosphate-buffered saline (PBS), and blocked with 5% bovine serum albumin containing 0.3% Triton X-100 (GenScript) for 1 h at room temperature. The tablets were incubated overnight at 4 °C with anti-p65 antibody (1:100, Bioworld, ref. BS1560), followed by washing with PBS and incubation with 546-conjugated Goat Anti-Rabbit IgG (1:400, Beyotime) for 2 h at room temperature. Nuclei were stained with 4′,6-diamidino-2-phenylindole for 20 min, and the slides were mounted and examined under a fluorescence microscope to visualize p65 nuclear translocation.

### Flow cytometry analysis

For surface marker analysis, cells were pre-incubated with FcR blocking antibody (FcR Blocking Reagent, Miltenyi Biotec) at 1 µg per 1 × 10^6^ cells in 100 µl PBS for 15 min at 4 °C to prevent nonspecific binding. Fluorescently conjugated antibodies or isotype controls were added at manufacturer-specified concentrations, and the cells were incubated for an additional 2 h at 4 °C. Following two washes with ice-cold PBS, the samples were analyzed using an Agilent-Novocyte flow cytometer, and the data were processed using NovoExpress software to quantify marker expression.

### Ethics statement

All animal experiments adhered to ethical guidelines and were approved by the Institutional Animal Care and Use Committee of the Nanjing University (IACUC-2210008).

### Animal study

Female BALB/c mice (6–8 weeks old) were obtained from Beijing Vital River Laboratory Animal Technology and housed under specific pathogen-free conditions at the Nanjing University Medical School. To establish an orthotopic breast cancer model, 4T1-luc cells were suspended in RPMI 1640 medium at a concentration of 1 × 10^7^ cells/ml, and 20 µl (2 × 10^5^ cells) was injected into the inguinal mammary fat pads of syngeneic mice. Mice were randomly assigned to three groups: PBS control, untransduced (UTD) control, and SR CAR-M treatment. On day 7 post-implantation, when the tumors became palpable, treatment was initiated with a single intravenous (i.v.) injection of 1 × 10⁶ DiR-labeled macrophages in 100 µl PBS. Tumor volumes were measured every third day using a Vernier caliper and calculated as 0.5 × length × width × width formula. The investigators were blinded to the group allocation during tumor measurements and subsequent analyses to minimize bias.

For the domain-deletion control in vivo study, mice were randomly assigned to five groups: PBS control, UTD BMDM, SR CAR-BM, ΔTLR9 SR CAR-M, and ΔIL-10R SR CAR-M. BMDMs were differentiated and transduced as described earlier. On day 7 post-tumor implantation, treatment was initiated with an i.v. injection of 1 × 10^6^ macrophages in 100 µl PBS, administered every 7 days for a total of three doses. Tumor volumes and body weights were monitored as described earlier. At the experimental end point (day 28), tumors and major organs were harvested for flow cytometric analysis of tumor-infiltrating immune cells, quantitative PCR (qPCR) analysis, serum biochemistry, organ weight measurement, and histopathological examination.

### Pro-invasion assay

To evaluate tumor cell invasion, 4T1 cells were mixed with macrophages at a 1:1 ratio and seeded into Matrigel-coated invasion chambers (24-well, 8 µm pore size) in RPMI 1640 medium. After 24 h of incubation at 37 °C, the cells were fixed with 4% paraformaldehyde, stained with crystal violet, and invading cells were quantified under a light microscope across five random fields per well.

### Cell cycle assay

4T1 cells co-cultured with SR CAR-M or control Raw264.7 macrophages were harvested, resuspended in 300 µl PBS, and fixed overnight in 700 µl pre-chilled absolute ethanol at −20 °C. Fixed cells were washed, resuspended in 500 µl PBS with 5 µl RNase, and incubated at 37 °C for 30 min to remove RNA. Cells were then stained with 5 µl propidium iodide (PI) for 30 min at room temperature, washed twice with ice-cold PBS, and analyzed by flow cytometry to determine the cell cycle distribution.

### Cell apoptosis assay

Apoptosis in 4T1 cells was assessed using the Beyotime Apoptosis Assay Kit. Harvested cells were stained with 100 µl of 1× binding buffer containing 5 µl Annexin V and 5 µl PI, incubated for 10 min at room temperature, and diluted with an additional 100 µl of 1× binding buffer. Samples were analyzed using flow cytometry to quantify early and late apoptotic populations.

### CCK-8 cell viability assay

Macrophage viability was measured using the Cell Counting Kit-8 (CCK-8) assay. Cells were seeded at 100 cells per well in 96-well plates with three replicates per group and cultured at 37 °C with 5% CO_2_. At designated time points (24, 48, 72, and 96 h), 50 µl DMEM containing 25 µl CCK-8 reagent was added to each well, followed by a 3-h incubation. Absorbance was measured at 450 nm using a microplate reader, and viability was calculated relative to untreated controls.

### In vitro cytotoxicity assay

The cytotoxic activity of SR CAR-Ms was independently validated using a lactate dehydrogenase (LDH) release assay. Tumor target cells (4T1, MB49, LLC, or B16) were seeded in 96-well plates, and SR CAR-M or UTD macrophages were added at effector-to-target (E:T) ratios of 1:1, 3:1, 5:1, and 8:1, with three replicates per condition. After 24 h of co-culture, the plates were centrifuged briefly, and cell-free supernatants were collected. LDH activity was measured using a commercial LDH cytotoxicity detection kit (C0017, Beyotime), according to the manufacturer’s instructions.

### SR CAR-Ms co-culture with M2

Monolayers of CAR-Ms and M2 macrophages were established separately for co-culture, as previously described. Briefly, SR CAR-Ms cultured on 25-mm-diameter Nunc plastic coverslips were activated with IL-10 (100 ng/ml). M2 macrophages were differentiated in 6-well plates. For co-culture, SR CAR-M-seeded coverslips were inverted onto confluent monolayers of M2 macrophages. After 24 h of contact, the phenotypic state of the M2 macrophages was evaluated.

### Bead-based phagocytosis assay

Phagocytic capacity was assessed using fluorescent red latex beads (1 µm diameter, L-2778; Sigma-Aldrich). Macrophages were prestimulated with or without 100 ng/ml IL-10 for 24 h. Beads were opsonized in complete medium (10% FBS in RPMI 1640) for 1 h at 37 °C, added to macrophages at a 10:1 ratio, and incubated for 4 h at 37 °C. Phagocytosis was halted with 1 ml ice-cold PBS, and the cells were washed thrice, harvested, and analyzed using flow cytometry to determine bead uptake.

### FACS-based phagocytosis assay

To measure tumor cell phagocytosis, macrophages were prestimulated with or without 100 ng/ml IL-10 for 24 h and co-cultured with 2 × 10^5^ DiR-labeled 4T1 cells for 6 h at E:T ratios of 1:1, 2:1, and 4:1. Cells were harvested, extensively washed, and stained with PE-labeled anti-CD11b to identify macrophages. Phagocytosis was quantified using flow cytometry, and the phagocytic index was calculated as the mean fluorescence intensity of DiR within CD11b⁺ cells.

### Construction of lentiviral vectors

The SR CAR cDNA was synthesized by Sangon Biotech, and the SR CAR gene was inserted into the pLenti6/v5 lentiviral vector using restriction enzymes SpeI, SacⅡ, and T4 ligase (TAKARA). Lentiviruses were produced in HEK293T cells by co-transfecting the target plasmid with two packaging plasmids (psPAX2 and pMD2G) using Lipofectamine 2000 (Invitrogen).

### Construction of adenoviral vectors

The SR CAR cassette was subcloned into an Ad5/F35 adenoviral vector (VectorBuilder). Murine and humanized SR CAR adenoviruses were packaged separately in HEK 293A cells using the jetPRIME transfection reagent (#24Y1711M7, Polyplus), according to the manufacturer’s instructions. Adenoviral particles were harvested 10–14 days post-transfection by three cycles of freeze–thaw lysis and purified by cesium chloride density gradient ultracentrifugation. Viral titers were determined using the Adeno-X Rapid Titer Kit (no. 632250, Takara). Two domain-deletion control constructs were generated in the same Ad5/F35 backbone: (1) ΔTLR9 SR CAR, retaining the signal peptide, HA-tag, IL-10Rα extracellular domain, and transmembrane domain but lacking the TLR9 intracellular signaling domain; and (2) ΔIL-10R SR CAR, in which the IL-10Rα extracellular domain was deleted. For the humanized construct, the murine IL-10Rα extracellular domain and TLR9 intracellular domain were replaced with the corresponding human sequences.

### Primary bone marrow-derived macrophage isolation, differentiation, and transduction

Bone marrow cells were harvested from the femurs of 6–8-week-old female C57BL/6J mice. Cells were counted and seeded at a density of 3–5 × 10⁷ cells per 15-cm dish in DMEM supplemented with 10% FBS, 100 U/ml penicillin–streptomycin, and 50 ng/ml recombinant murine M-CSF (PeproTech). On day 4 of differentiation, SR CAR, ΔTLR9 SR CAR, or ΔIL-10R SR CAR adenovirus was added to the culture at a multiplicity of infection of 1000. After 24 h, the medium was replaced with fresh complete DMEM containing 50 ng/ml M-CSF. Transduction efficiency was assessed by flow cytometric analysis of HA-tag expression.

### Mature BMDC differentiation

Bone marrow-derived dendritic cells (BMDCs) were generated following established protocols (Xia Zhang, Ricardo Goncalves, and David M. Mosser). Bone marrow cells from BALB/c mice were cultured in 24-well plates containing RPMI 1640 medium with 10% FBS, 20 ng/ml granulocyte–macrophage colony-stimulating factor (GM-CSF), and 20 ng/ml IL-4 for 6 days. Suspended cells were harvested, reseeded in 6-well plates at 1 × 10^6^ cells/ml, and stimulated with 20 ng/ml GM-CSF and 20 ng/ml IL-4 for an additional 2 days to obtain mature BMDCs.

### Human monocyte-derived macrophage isolation, differentiation, and transduction

Human peripheral blood was obtained from MILECELL BIO. (Shanghai, China). All fresh whole blood samples were collected from healthy volunteers with informed consent. Peripheral blood mononuclear cells (PBMCs) were isolated by density gradient centrifugation using Ficoll lymphocyte separation medium (density 1.077 g/ml; 40503ES76, Yeasen). CD14⁺ monocytes were positively selected using CD14 MicroBeads (130-050-201, Miltenyi Biotec) according to the manufacturer’s protocol. Purified CD14⁺ monocytes were cultured in X-VIVO 15 serum-free medium (04-418Q, Lonza) supplemented with 10% FBS, 100 U/ml penicillin–streptomycin, and 50 ng/ml recombinant human M-CSF (Z02924, GenScript) at 37 °C in a humidified atmosphere with 5% CO_2_. On day 5 of macrophage differentiation, cells were transduced with humanized SR CAR adenovirus at a multiplicity of infection of 1,000. After 24 h of incubation, the medium was replaced with fresh complete medium, yielding human primary SR CAR-Mac for subsequent functional experiments.

### T cell proliferation assay

T cell proliferation was assessed using spleen-derived T cells. Anti-mouse CD3e monoclonal antibody (Functional Grade 70-AM003E-100) was diluted to a concentration of 5 μg/ml with completed DMEM, seeded at 50 μl per well in 96-well plate, and incubated at 4 °C overnight. Mouse primary T cells were isolated from the spleen using a magnetic bead sorting kit (Stem Cell no. 19851), and the T cells were stained with carboxyfluorescein succinimidyl ester (CFSE) for 15 min at 37 °C. About 4 × 10^4^ macrophages and 4 × 10^5^ CFSE-labeled primary T cells were co-cultured in the CD3 antibody pre-incubated in 96 wells plate for 3 days, simultaneously added with 5 μg/ml anti-mouse CD28 monoclonal antibody (Functional Grade 70-AM028-100), and macrophages in some wells were stimulated with 100 ng/ml IL-10 consistent with the group’s design. T cell proliferation rate was measured using flow cytometry.

### siRNA-mediated MyD88 knockdown

Three small interfering RNA (siRNA) duplexes targeting murine MyD88 were designed and synthesized by GenScript. The sequences are listed in Supplementary Table 3. A non-targeting scrambled siRNA (GenScript) was used as a negative control. SR CAR-M cells (Raw264.7-derived) were seeded at a density of 3 × 10^5^ cells per well in 6-well plates and transfected with 30 nM siRNA using Lipofectamine RNAiMAX (Invitrogen), following the manufacturer’s instructions. The transfection medium was replaced with complete DMEM after 6 h. Knockdown efficiency was assessed 48 h post-transfection by western blot analysis of MyD88 protein expression.

### RT-qPCR, western blotting, and ELISA

Gene expression, protein levels, and cytokine secretion were analyzed using standard protocols, as previously described^29^. The primers used for RT-qPCR are listed in Supplementary Tables 1 and 2. Western blotting was performed using antibodies against STAT3, phospho-STAT3, IRF1, IκBα, phospho-IκBα, MyD88, and p65, with β-actin as the loading control. IL-10 levels in the culture supernatants were quantified using enzyme-linked immunosorbent assay (ELISA) with a commercial kit (Beyotime). IL-6 and TNF-α levels in the supernatants of MyD88 knockdown experiments were quantified using mouse IL-6 and TNF-α ELISA kits (Beyotime), respectively. Serum IL-10 concentrations in tumor-bearing mice were measured at days 7, 14, and 21 post-tumor implantation using the same IL-10 ELISA kit. Blood was collected from the retro-orbital sinus, allowed to clot for 30 min at room temperature, and serum was separated by centrifugation at 3,000×*g* for 15 min at 4 °C.

### Statistical analysis

All statistical analyses were performed using GraphPad Prism 9 software (GraphPad). Differences between the two groups were calculated using the Student’s *t* test. Differences among multiple groups were calculated using one-way or two-way analysis of variance. Differences between the tumor growth curves were determined using one-way analysis of variance. Statistical significance was set at *P* ≤ 0.05. For all figures, *P < 0.05, **P < 0.01, and ***P < 0.001. All data are shown as mean ± SEM, unless noted otherwise in the figure legend. All experiments were performed with *n* ≥ 3 biological replicates.

## Results

### Generation and characterization of SR CAR-Ms

TAMs, particularly those with the M2-like phenotype, have a pivotal role in shaping immunosuppression within tumors through the secretion of factors, such as IL-10. To investigate the clinical relevance of IL-10 in cancer, we analyzed data from The Cancer Genome Atlas and GTEx databases for 33 cancer types. IL-10 expression was elevated in several malignancies, including diff use large B-cell lymphoma (DLBC), glioblastoma multiforme (GBM), and ovarian serous cystadenocarcinoma (OV), compared with that in normal tissues (Supplementary Fig. 1a,b). Kaplan–Meier survival analysis revealed that high IL-10 expression correlated with poor overall survival across multiple cancers (Supplementary Fig. 1c–e). In breast invasive carcinoma (BRCA), IL-10 expression was positively correlated with immune, stromal, and ESTIMATE scores (Supplementary Fig. 1f,g), as well as with the infiltration of macrophages and regulatory T cells (Supplementary Fig. 1h,i). These findings suggest that targeting IL-10 signaling may reprogram the TME.

**Fig. 1:**
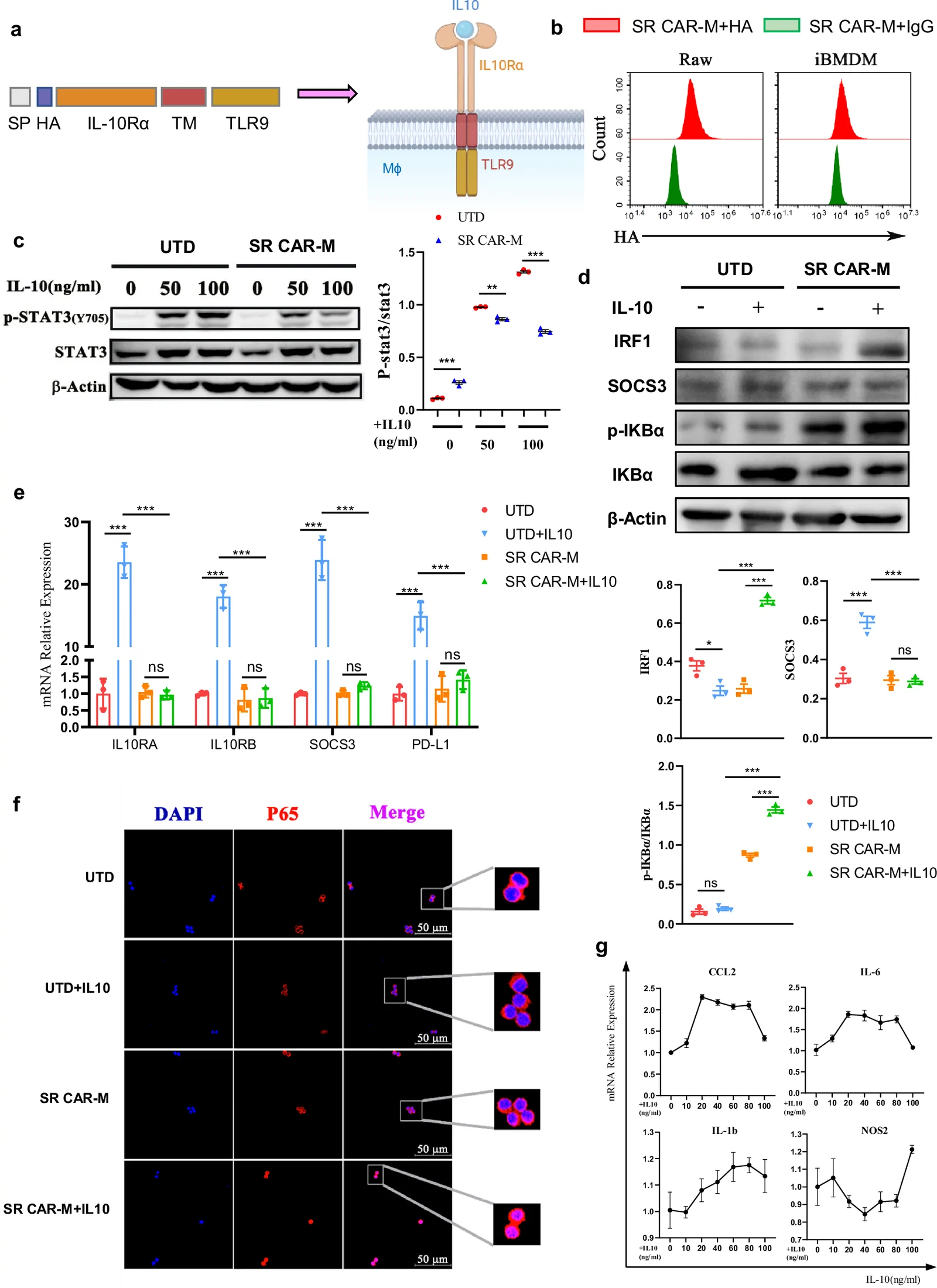
Generation and characterization of SR CAR-M. **a** Schematic representation of the SR CAR construct design for macrophages. **b** Representative flow cytometric analysis of transduction efficiency (HA-tag expression) in macrophages (Raw264.7 cells and iBMDM cells) 2 days after transduction with SR CAR. **c** Western blot analysis of IL-10 signaling pathways STAT3 and p-STAT3 on SR CAR-M or UTD in response to different concentrations of IL-10, and their grayscale statistical analysis is shown (right). **d** The TLR9 signaling pathways IKBα and p-IKBα and IL-10 signaling pathways IRF1 and SOCS3 on SR CAR-M or UTD in response to IL-10 stimulation were detected by western blot analysis, and their grayscale statistical analysis is shown at (bottom). **e** The mRNA expression levels of IL-10RA, IL-10RB, SOCS3, and PD-L1 in the IL-10 signaling pathway were examined using quantitative PCR (qPCR). **f** The intracellular distribution of the NF-kB subunit P65 (40×) in SR CAR-M cells after stimulation with IL-10 (100 ng/ml) for 6 h was detected using immunofluorescence. **g** Transcription levels of TLR9 downstream target genes, including CCL2, IL-6, IL-1B, and NOS2, after stimulation with concentration gradient IL-10 (0–100 ng/ml) for 1 day, were detected by qPCR. All values are expressed as mean ± SEM from *n* = 3 independent biological replicates, with each biological replicate performed in technical triplicate. *P < 0.05, **P < 0.01, and ***P < 0.001. *DAPI* 4′,6-diamidino-2-phenylindole, *iBMDM* immortalized bone marrow-derived macrophage, *ns* not significant, *SR* *CAR*-*M* switch receptor chimeric antigen receptor-macrophage, *STAT3* signal transducer and activator of transcription factor 3, *TLR* Toll-like receptor, *UTD* untransduced.

To target IL-10 and achieve redirection of the macrophage phenotype, we generated a CAR-like switch receptor construct (SR CAR) comprising the IL-10 receptor α subunit (IL-10Rα) extracellular domain fused to the TLR9 intracellular signaling domain (Fig. 1a). This unique SR CAR design allows the IL-10Rα extracellular domain to specifically bind IL-10, triggering a signal switch toward a pro-inflammatory response by connecting to the TLR9 domain. We then engineered an SR CAR into a lentivirus vector and demonstrated that lentivirus-transduced Raw264.7 and immortalized BMDMs (iBMDMs) expressed SR CAR efficiently (Fig. 1b). Unless otherwise specified, all subsequent in vitro and in vivo experiments were performed using SR CAR-transduced Raw264.7 macrophages, hereafter referred to as SR CAR-M.

**Fig. 2:**
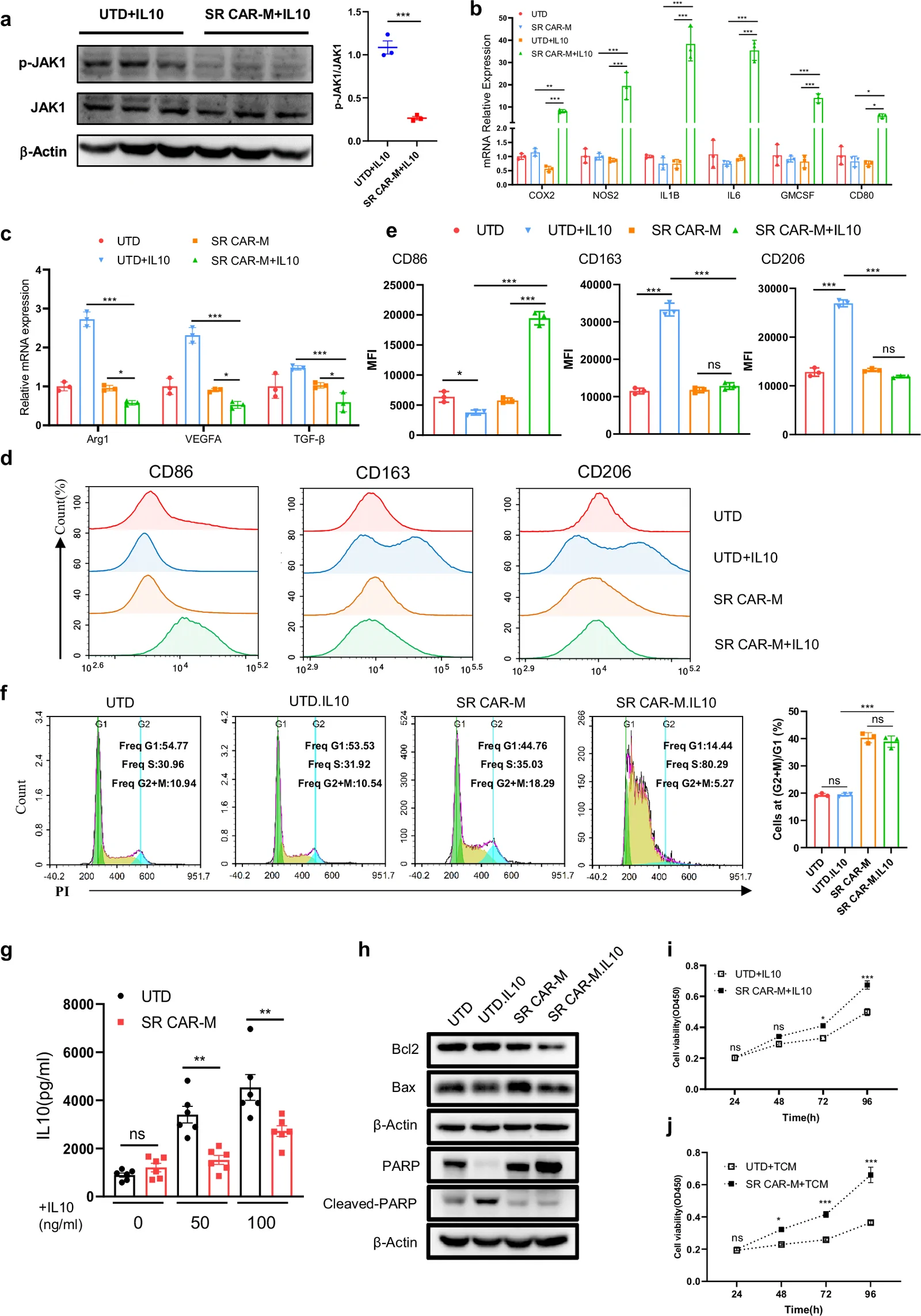
IL-10 induced M1-like phenotype on SR CAR-Ms. **a** The IL-10 signaling pathways JAK1 and p-JAK1 on SR CAR-M or UTD in response to IL-10 stimulation were detected by western blot analysis, and their grayscale statistical analysis is shown (right). Transcription levels of pro-inflammatory (M1) genes (part **b**) and anti-inflammatory (M2) genes (part **c**) on SR CAR-M or UTD in response to IL-10 were detected by quantitative PCR after IL-10 (50 ng/ml) stimulation for 1 day. **d** Representative flow cytometric analysis of M1 (CD86) and M2 (CD163 and CD206) markers. **e** Mean fluorescence intensity (MFI) of the markers. **f** Cell cycle analysis of SR CAR-M and UTD after stimulation with IL-10 for 2 days by flow cytometry. **g** SR CAR-M and UTD were treated with different concentrations of IL-10 for 48 h. The IL-10 concentration in the culture medium was detected using an enzyme-linked immunosorbent assay. **h** SR CAR-M and UTD were harvested after stimulation with IL-10 for 2 days and assessed for the expression of apoptosis-related proteins using western blotting. The cell viability of SR CAR-M and UTD after co-culture with IL-10 (part **i**) and 30% tumor-conditioned medium (TCM) (part **j**) was detected by the CCK-8 assay. All values are expressed as mean ± SEM from *n* = 3 independent biological replicates, with each biological replicate performed in technical triplicate (except part **g**, which is *n* = 5). *P < 0.05, **P < 0.01, and ***P < 0.001. *GM*-*CSF* granulocyte–macrophage colony-stimulating factor, *JAK* Janus kinase, *ns* not significant, *PARP* poly (ADP-ribose) polymerase, *PI* propidium iodide, *SR* *CAR*-*M* switch receptor chimeric antigen receptor-macrophage, *TGF*-*β* transforming growth factor-beta, *UTD* untransduced, *VEGFA* vascular endothelial growth factor A.

Next, we examined whether SR CAR converted IL-10 into a pro-inflammatory signal activated by TLR9. IL-10 signal blocking in macrophages was evaluated by the degree of phosphorylation of Janus kinase (p-JAK) and signal transducer and activator of transcription factor 3 (p-STAT3), the downstream transcription factors activated by IL-10. SR CAR-Ms exhibited markedly reduced p-JAK1 and p-STAT3 expression compared with UTD-M following IL-10 stimulation (Figs. 2a and 1c). In addition, SR CAR-Ms maintained expression of IL-10 receptor components (IL-10RA and IL-10RB) while downregulating SOCS3, a negative regulator of cytokine signaling, and the immunosuppressive marker PD-L1, following IL-10 stimulation (Fig. 1d,e). Conversely, IL-10 stimulation activated TLR9 downstream signaling in SR CAR-Ms, as evidenced by increased IκBα phosphorylation, upregulation of IRF1, and nuclear translocation of NF-κB p65 (Fig. 1d,f). To confirm that this signaling depends on canonical TLR9 adaptor machinery, we knocked down MyD88 in SR CAR-M cells using siRNA (Supplementary Fig. 2a). MyD88 silencing abolished NF-κB p65 nuclear translocation (Supplementary Fig. 2b), markedly reduced IL-6 and TNF-α secretion (Supplementary Fig. 2c), and suppressed transcription of NOS2, IL-1β, and IL-6 (Supplementary Fig. 2d), demonstrating that the SR CAR signals through MyD88-dependent pathways. A key question is whether this signaling requires endosomal trafficking, as canonical TLR9 activation depends on endosomal acidification and CpG DNA recognition. To address this, we pretreated SR CAR-M cells with chloroquine, a well-characterized inhibitor of endosomal acidification that blocks canonical TLR9 signaling. Chloroquine treatment did not impair NF-κB p65 nuclear translocation (Supplementary Fig. 2b) nor suppress pro-inflammatory gene transcription (Supplementary Fig. 2d), indicating that SR CAR-mediated signaling operates at the cell surface independently of endosomal processing. Together, these findings establish that the SR CAR engages canonical MyD88-dependent downstream signaling but is activated through a non-canonical, endosome-independent mechanism, consistent with IL-10-induced receptor oligomerization bringing TLR9 intracellular domains into proximity for MyD88 recruitment at the plasma membrane.

To assess the dynamic range of SR CAR responsiveness, we stimulated macrophages with IL-10 across a concentration gradient (0–100 ng/ml). Target gene expression displayed dose-dependent responses, with genes such as CCL2 and IL-6 reaching peak transcription at 20 ng/ml IL-10, whereas higher concentrations (100 ng/ml) suppressed their expression (Fig. 1g). These findings demonstrate that SR CAR successfully converts immunosuppressive IL-10 signals into pro-inflammatory TLR9 responses in macrophages in a concentration-dependent manner.

To rigorously dissect the functional contributions of each receptor component, we generated two domain-deletion controls in primary BMDMs: ΔTLR9 SR CAR-M (IL-10Rα extracellular domain only, lacking TLR9 signaling) and ΔIL-10R SR CAR-M (TLR9 intracellular domain only, lacking IL-10 binding). All constructs achieved efficient transduction (Supplementary Fig. 3a). Upon IL-10 stimulation, only the full-length SR CAR-BM exhibited the complete signal-switching profile: STAT3 suppression coupled with IRF1 upregulation, IκBα phosphorylation, and NF-κB nuclear translocation (Supplementary Fig. 3b,c). ΔTLR9 SR CAR-M sequestered IL-10 (suppressing p-STAT3) but failed to activate TLR9 signaling, whereas ΔIL-10R SR CAR-M showed neither STAT3 suppression nor TLR9 activation. Accordingly, only SR CAR-BM maintained expression of IL-10 receptor components (IL-10RA and IL-10RB) while downregulating SOCS3 and PD-L1, following IL-10 stimulation (Supplementary Fig. 3d), confirming that both receptor components are indispensable for signal switching. These primary BMDM results also validated our Raw264.7 findings in a physiologically relevant cell type.

To establish translational relevance, we engineered a humanized SR CAR by replacing the murine IL-10Rα and TLR9 domains with their human orthologs and transduced PBMC-derived macrophages using an Ad5F35 adenoviral vector (Supplementary Fig. 4a,b). Human SR CAR-Mac recapitulated the murine phenotype: STAT3 suppression, IκBα phosphorylation, IRF1 upregulation (Supplementary Fig. 4c), SOCS3 and PD-L1 downregulation (Supplementary Fig. 4d), and NF-κB nuclear translocation (Supplementary Fig. 4e). These results confirm the cross-species conservation of the signal-switching mechanism.

### SR CAR-Ms exhibited an M1-like phenotype and enhanced viability in response to IL-10

Next, we investigated whether signal switching alters the macrophage phenotype. Consistent with TLR9 pathway activation, IL-10 stimulation of SR CAR-Ms resulted in the upregulation of M1-associated markers, including COX2, NOS2, IL-1β, IL-6, GM-CSF, CD80, and CD86, and downregulation of M2-associated markers, including Arg1, VEGFA, TGF-β, CD163, and CD206 (Fig. 2b–e). Importantly, this IL-10-driven M1 polarization was not restricted to Raw264.7 cells. Primary SR CAR-BM and human SR CAR-Mac similarly exhibited upregulation of M1-associated genes and downregulation of M2-associated markers upon IL-10 stimulation (Supplementary Fig. 3e,f and Supplementary Fig. 4f–h), demonstrating that the signal-switching mechanism confers a consistent M1-polarizing capacity across distinct macrophage sources and species. Thus, IL-10, which normally promotes immunosuppression, paradoxically drove SR CAR-Ms toward an immunostimulatory M1-like state. Supporting active IL-10 internalization through the switch receptor, quantification of culture supernatants revealed dose-dependent IL-10 consumption by SR CAR-Ms, with maximal consumption at physiologically relevant concentrations (50 ng/ml) (Fig. 2g), suggesting that SR CAR-Ms function as IL-10 sinks within tumor tissues.

Given that genetic engineering can impose a metabolic burden, we assessed whether SR CAR expression affected macrophage viability under inflammatory conditions. Cell cycle analysis revealed that IL-10 stimulation increased the proportion of SR CAR-Ms in the G2/M phase compared with UTD-Ms (Fig. 2f), which indicates an enhanced proliferative capacity. Although IL-10 induced PARP cleavage in UTD-Ms, SR CAR-Ms resisted IL-10-mediated apoptosis and maintained high PARP expression (Fig. 2h). We further evaluated cell viability under TME conditions by culturing the macrophages in tumor-conditioned medium (TCM). SR CAR-Ms demonstrated significantly enhanced viability compared with UTD-Ms in both IL-10-supplemented medium and TCM over 96 h (Fig. 2i,j).

**Fig. 3:**
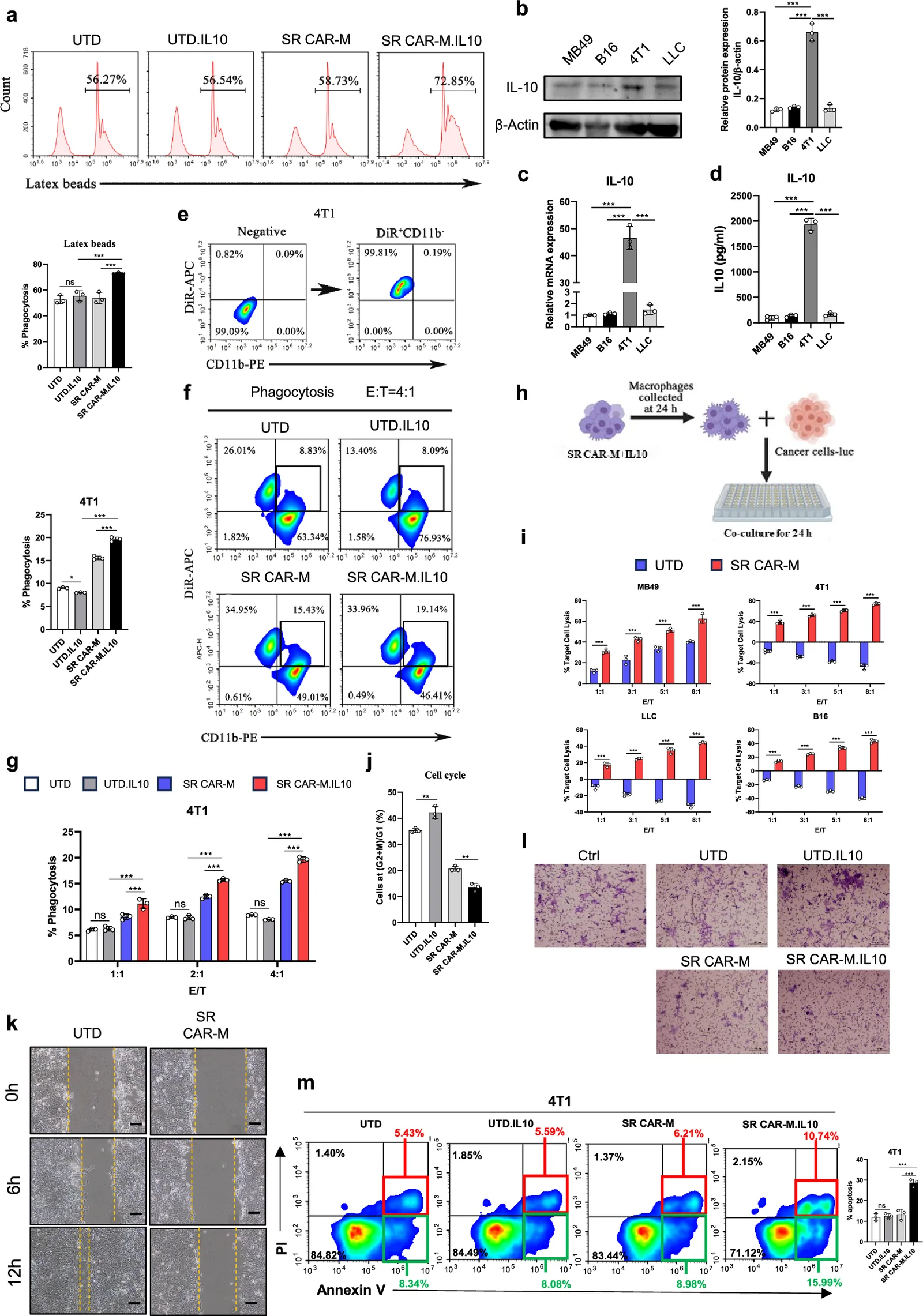
SR CAR-M cells showed target-dependent phagocytosis and anticancer cell functions in vitro and had effects on apoptosis, proliferation, and invasion of cancer cells. **a** Phagocytosis of latex beads by UTD, UTD.IL-10, SR CAR-M, or SR CAR-M.IL-10 cells. Protein (part **b**) and mRNA expression (part **c**) of IL-10 in MB49, B16, 4T1, and LLC cells. **d** Cytokine (IL-10) secretion of the MB49, B16, 4T1 and LLC cells detected by an enzyme-linked immunosorbent assay. **e** Tumor cells were labeled with DiR. **f** Phagocytosis of UTD, UTD.IL-10, SR CAR-M, and SR CAR-M.IL-10 cells were detected using flow cytometry. **g** Statistical analysis of phagocytosis detection experiments. **h** Graphical schema of the experimental design to detect the cell-killing ability of UTD and SR CAR-M cells. **i** Killing assay of multiple tumor cells by UTD and SR CAR-M cells at different E:T ratios (macrophage: tumor = 1:1, 3:1, 5:1, 8:1), including breast, bladder, lung, and melanoma cancers. **j** Cell cycle analysis of 4T1 cells after co-culture with UTD or SR CAR-M cells. **k** A wound-healing assay was performed to evaluate the cell migration ability of 4T1 cells co-cultured with UTD or SR CAR-M. Scale bar, 100 μm. **l**, An invasion assay with 4T1 cells after co-culture with UTD or SR CAR-M cells was evaluated using crystal violet staining. Scale bar, 100 μM. **m**, Flow cytometry analysis of apoptosis in 4T1 cells after co-culture with UTD or SR CAR-M cells via annexin V/PI staining. All values are expressed as mean ± SEM from *n* = 3 independent biological replicates, with each biological replicate performed in technical triplicate. *P < 0.05, **P < 0.01, and ***P < 0.001. *ns* not significant, *PI* propidium iodide, *SR* *CAR*-*M* switch receptor chimeric antigen receptor-macrophage, *UTD* untransduced.

The TME presents a complex milieu of immunosuppressive signals that typically override M1 programming. To test phenotypic stability, we co-cultured IL-10-treated UTD (UTD.IL-10) and SR CAR-M (SR CAR-M.IL-10) cells with 4T1 tumor cells that secrete large amounts of IL-10 (Fig. 3d). SR CAR-M.IL-10 robustly maintained M1 markers (CD80, CD86, major histocompatibility complex (MHC) II, and CD40) while resisting upregulation of M2-associated markers (CD206), whereas UTD-Ms adopted an M2-like phenotype (Supplementary Fig. 5a). We subjected UTD.IL-10 and SR CAR-M.IL-10 to a more stringent challenge by combining IL-4 and IL-13, which are canonical M2-inducing cytokines (Supplementary Fig. 5b). Remarkably, SR CAR-M.IL-10 sustained high-level expression of M1 markers, including NOS2, IL-1β, IL-6, TNF-α, CD80, CD86, and MHC II, and blocked the induction of M2-associated markers, including Arg1, TGF-β, CD163, and CD206, whereas UTD-Ms underwent complete M2 polarization under identical conditions (Supplementary Fig. 5c,d). Furthermore, UTD-Ms exposed to TCM upregulated a prototypical M2 transcriptional program, including VEGFA, TGFβ, Arg1, and MMP9 (Supplementary Fig. 6a). By contrast, TCM-treated SR CAR-Ms showed M1 phenotypes, as evidenced by the reduced expression of M2-associated genes and enhanced expression of M1 markers (Supplementary Fig. 6b). To determine whether this stability represents a genuine advantage over conventional polarization, we directly compared SR CAR-Ms with LPS/IFNγ-polarized M1 macrophages after 48-h TCM challenge. Conventionally polarized macrophages lost M1 markers and acquired M2 gene expression, whereas SR CAR-Ms maintained their phenotype throughout (Supplementary Fig. 5e), demonstrating that continuous IL-10 in the TME actively reinforces the M1 state through sustained TLR9 activation.

### SR CAR-Ms exhibited enhanced phagocytosis and antitumor cytotoxicity

To detect the potential for SR CAR-mediated redirection of macrophage phagocytosis, we first measured the phagocytic capacity of UTD and SR CAR-M cells using fluorescent latex beads as surrogate targets. SR CAR-M.IL-10 exhibited markedly elevated bead uptake compared with that of UTD-M and UTD-M.IL-10 and SR CAR-M (Fig. 3a).

A critical consideration was whether the tumor cells themselves could provide the IL-10 necessary to activate SR CAR-Ms. We examined IL-10 expression across a panel of murine tumor cell lines, including MB49 bladder carcinoma, B16 melanoma, 4T1 breast cancer, and LLC lung cancer. Among the four tumor lines, 4T1 cells exhibited particularly robust expression at both the protein and transcript levels (Fig. 3b,c). Importantly, 4T1 tumor cells actively secreted IL-10 into the extracellular milieu at concentrations of up to 2000 pg/ml (Fig. 3d). This finding revealed a potential therapeutic vulnerability: tumors produce IL-10 to establish immunosuppression; however, this same IL-10 could serve as an activation signal for SR CAR-Ms, effectively converting a tumor defense mechanism into a liability.

We labeled 4T1 cells with DiR and incubated them with UTD and SR CAR-M (Fig. 3e). Compared with UTD and UTD.IL-10, SR CAR-M and SR CAR-M.IL-10 showed increased phagocytic activity against 4T1 cells at all effector-to-target ratios examined (Fig. 3f,g). Notably, SR CAR-M mediated potent killing across all cell lines tested, with cytotoxicity increasing proportionally with the effector-to-target ratio (Fig. 3h,i), which may be attributed to tumor cell-secreted IL-10 activating of the switch receptor. Moreover, it is worth noting that SR CAR-M showed broad-spectrum antitumor activity independent of tumor lineage or antigen expression. Notably, the enhanced phagocytosis and cytotoxicity were not restricted to Raw264.7 cells: primary SR CAR-BM and human SR CAR-Mac also exhibited significantly augmented effector functions against tumor cells (Supplementary Fig. 3g,h and Supplementary Fig. 4i–k), indicating that these functional outputs are intrinsic to the signal-switching receptor design rather than dependent on a specific cellular context. Conversely, neither ΔTLR9 SR CAR-M nor ΔIL-10R SR CAR-M enhanced phagocytosis or cytotoxicity (Supplementary Fig. 3g,h), confirming that these functional outputs require the integrated signal-switching mechanism. Co-culture with SR CAR-Ms induced substantial G2/M cell cycle arrest in 4T1 breast cancer cells (Fig. 3j), indicating the blockade of tumor cell division. Additionally, SR CAR-Ms markedly suppressed 4T1 cell migration and invasion compared with UTD-M treatment (Fig. 3k,l). SR CAR-Ms induced pronounced tumor cell apoptosis, with substantial increases in both early and late apoptotic populations compared with UTD treatment (Fig. 3m). Collectively, these findings demonstrate that SR CAR-Ms exert multifaceted antitumor effects, including enhanced phagocytosis, direct cytotoxicity, cell cycle arrest, suppression of migration and invasion, and induction of apoptosis.

### SR CAR-Ms promoted dendritic cell maturation and enhanced T cell proliferation and effector function

**Fig. 4:**
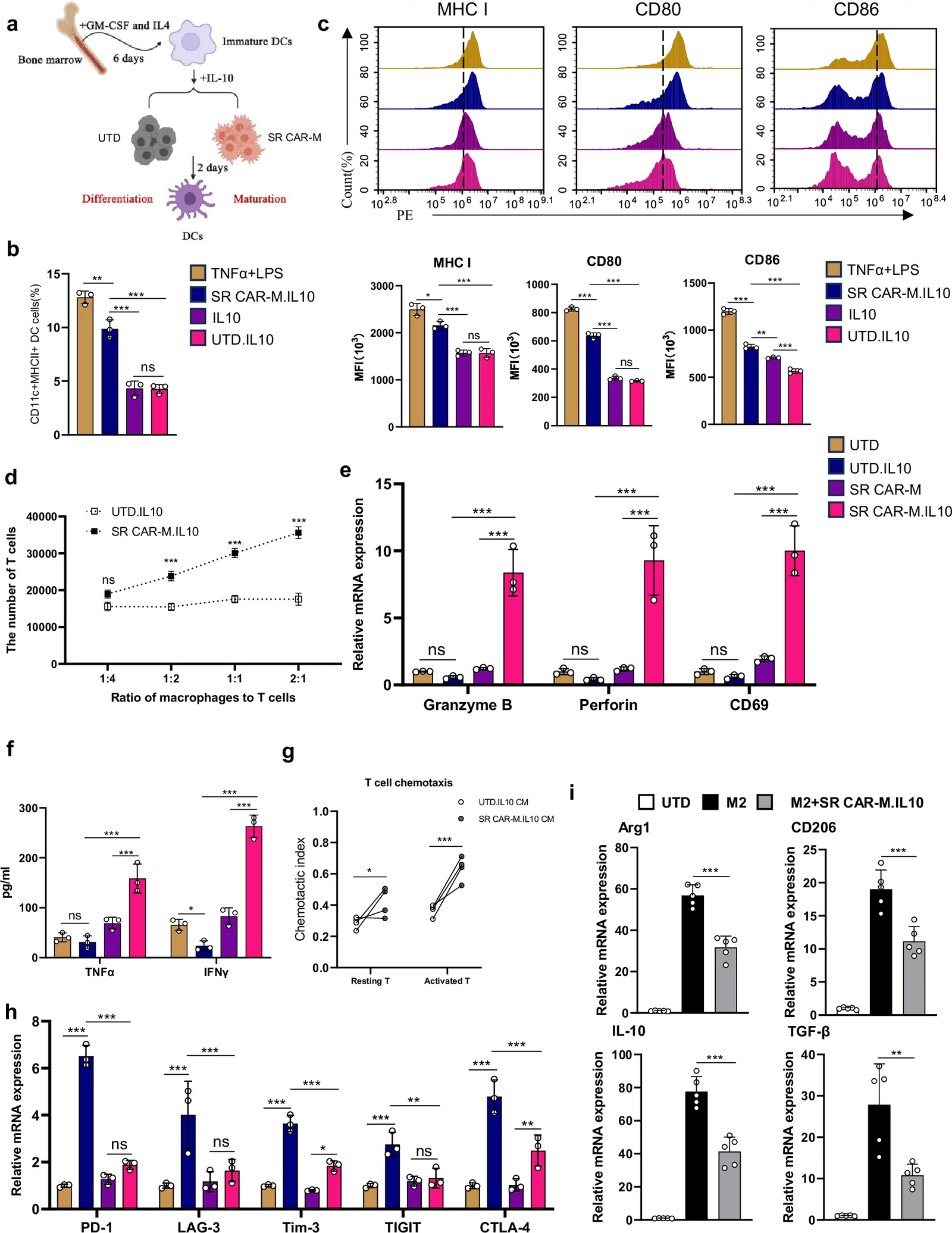
SR CAR-M induces differentiation and maturation of bone marrow-derived dendritic cells and enhances T cell proliferation in vitro. **a** Schematic diagram of the process of bone marrow-derived dendritic cells (BMDCs) differentiation in vitro. **b** Flow cytometry was used to detect the differentiation efficiency of BMDCs (CD11c+MHC II+) under different induction conditions. **c** The levels of BMDC maturation markers (MHC I, CD80, and CD86) were detected by flow cytometry under different induction conditions. **d** The proliferation of T cells after co-culture with UTD or SR CAR-M stimulated by IL-10 for 3 days. **e** Quantification of T cell activation markers by quantitative PCR (qPCR) after co-culture with UTD, UTD.IL-10, SR CAR-M or SR CAR-M.IL-10. **f** Measurement of inflammatory cytokine secretion from T cells after co-culture with UTD, UTD.IL-10, SR CAR-M, or SR CAR-M.IL-10 via enzyme-linked immunosorbent assay. **g** Chemotaxis of resting or activated T cells by UTD or SR CAR-M conditioned media. **h** Quantification of T cell exhaustion markers by qPCR in T cells after co-culture with UTD, UTD.IL-10, SR CAR-M, or SR CAR-M.IL-10. **i** M2 macrophages were co-cultured with IL-10-activated SR CAR-Ms for 24 h. The mRNA expression of Arg1, CD206, IL-10, and TGF-β in M2 was examined using qPCR. All values are expressed as mean ± SEM from *n* = 3 independent biological replicates, with each biological replicate performed in technical triplicate (except part **i** which is *n* = 5). *P < 0.05, **P < 0.01, and ***P < 0.001. *DC* dendritic cell, *GM*-*CSF* granulocyte–macrophage colony-stimulating factor, *IFN* interferon, *LPS* lipopolysaccharide, *MFI* mean fluorescence intensity, *MHC* major histocompatibility complex, *ns* not significant, *SR* *CAR*-*M* switch receptor chimeric antigen receptor-macrophage, *TGF*-*β* transforming growth factor-beta, *TNF* tumor necrosis factor, *UTD* untransduced.

DCs are professional antigen-presenting cells that bridge innate and adaptive immunity; however, IL-10 potently suppresses DC maturation and antigen presentation. We differentiated bone marrow-derived DCs with GM-CSF and IL-4 and then exposed immature DCs to IL-10 in the presence or absence of UTD/SR CAR-M (Fig. 4a). IL-10 suppressed DC differentiation, reducing the proportion of CD11c⁺MHC II⁺ mature DCs to ~4% compared with 13% with TNF-α and LPS stimulation, a classic combination for inducing DC maturation (Fig. 4b). Co-culture with SR CAR-M.IL-10 restored DC differentiation efficiency to levels comparable to those observed after TNF-α and LPS treatment (Fig. 4b), as evidenced by the elevated expression of maturation markers, including MHC I, CD80, and CD86 (Fig. 4c). By contrast, UTD-M.IL-10 failed to rescue DC maturation in this study. Consistent with the results obtained in Raw264.7 cells, primary SR CAR-BM similarly promoted DC maturation to comparable levels. By contrast, neither ΔTLR9 SR CAR-M nor ΔIL-10R SR CAR-M was able to rescue DC maturation under IL-10 suppression (Supplementary Fig. 8a), further demonstrating that the DC-activating capacity of SR CAR-Ms is uniquely attributable to the integrated signal-switching mechanism rather than IL-10 sequestration alone.

Interestingly, IL-10 alone enhanced T cell expansion (Supplementary Fig. 9a), consistent with the ability of IL-10 to support CD8⁺ T cell survival and proliferation, as reported in a clinical trial. Compared with UTD.IL-10, co-culture with SR CAR-M.IL-10 increased T cell proliferation in the absence of IL-10 (Fig. 4d). This enhanced proliferation was accompanied by the functional activation of SR CAR-M. IL-10 induced the upregulation of cytotoxic effector molecules, including granzyme B, perforin, and CD69, in T cells (Fig. 4e), which corresponded to the elevated secretion of TNF-α and IFNɣ (Fig. 4f). Additionally, an in vitro chemotaxis assay demonstrated that SR CAR-M.IL-10 cells directly induced the recruitment of both resting and activated murine T cells (Fig. 4g).

T cell exhaustion, characterized by the upregulation of inhibitory receptors, represents a major barrier to antitumor immunity. UTD-M.IL-10 induced the expression of exhaustion markers, including PD-1, LAG-3, Tim-3, TIGIT, and CTLA-4, in co-cultured T cells, whereas SR CAR-M.IL-10 suppressed these inhibitory receptors (Fig. 4h). The immunostimulatory function of SR CAR-M was assessed through co-culture experiments with M2 macrophages. SR CAR-M.IL-10 significantly deactivated M2 macrophages (Fig. 4I). Collectively, these findings demonstrate that SR CAR-M converted an immunosuppressive signal into a pro-inflammatory signal and orchestrated a coordinated antitumor immune response, despite the presence of IL-10.

### SR CAR-Ms infiltrate tumors, suppress tumor growth, and prolong tumor-bearing mice survival without systemic toxicity

**Fig. 5:**
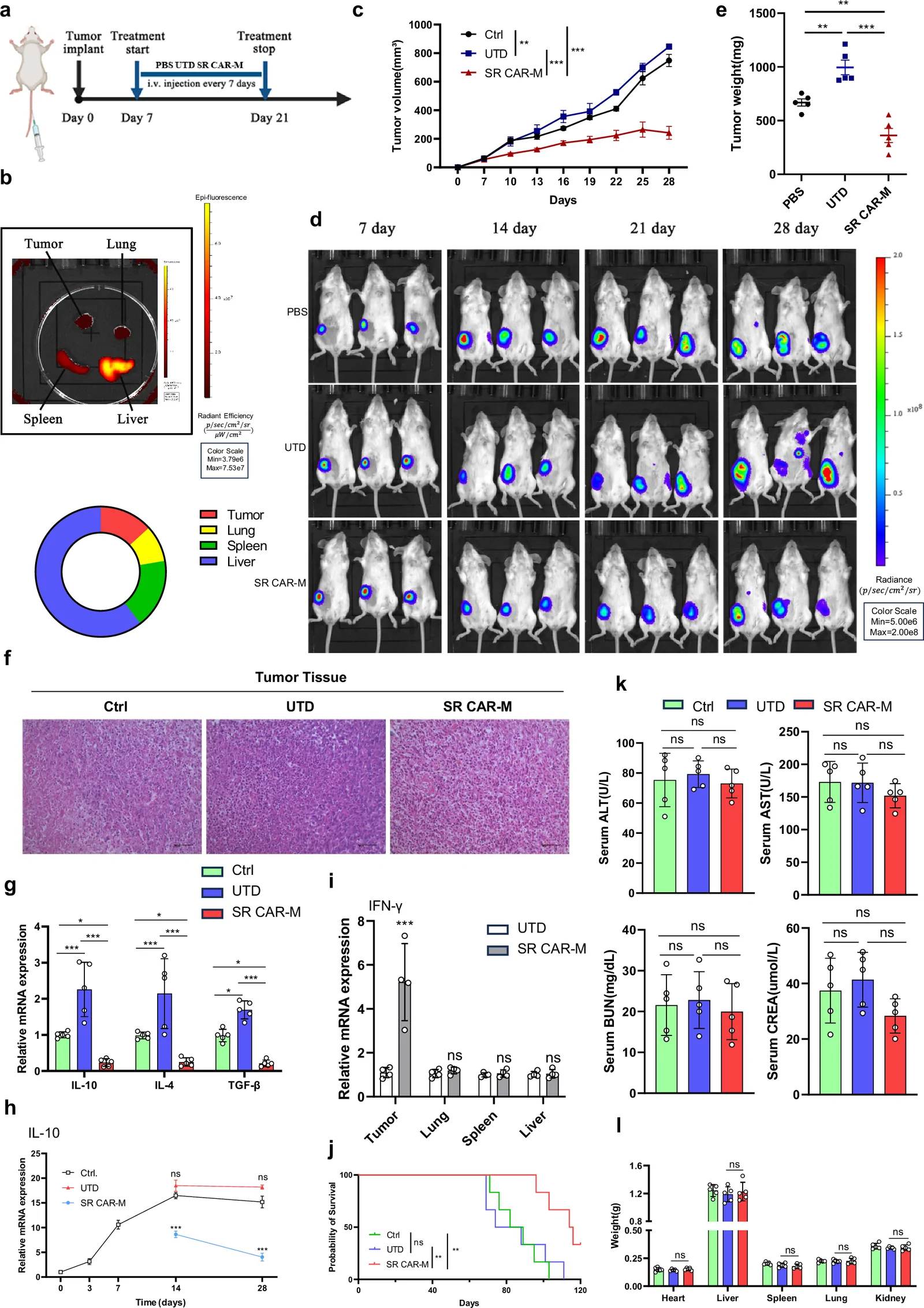
SR CAR-M effectively infiltrates tumor lesions and suppresses tumor growth in mice. **a** Experimental schedule for tumor implantation and macrophage infusion. **b** Representative images of DiR-labeled SR CAR-Ms accumulation in different tissues. **c** Tumor growth in mice from the control, UTD, and SR CAR-M groups was analyzed by measuring tumor volume every 3 days. **d** Bioluminescence imaging (BLI) was performed every 7 days to assess tumor burden. **e** Tumor weights in BALB/c mice treated with PBS, UTD, and SR CAR-Ms. **f** Tumor tissue samples from the Ctrl, UTD, and SR CAR-M groups were stained with hematoxylin and eosin. Scale bar, 50 mm. **g** Quantification of M2 markers by quantitative PCR in macrophages isolated from the tumor of mice in Ctrl, UTD, and SR CAR-M groups. **h** mRNA level of IL-10 in the tumors over the course of treatment. **i** UTD and CAR-Ms were sorted from various organs 72 h after injection. The M1-like phenotype of macrophages from various organs was assessed using quantitative PCR. **j** Survival analysis of nice in Ctrl, UTD, and SR CAR-M groups. **k** Analysis of serum biomarkers for liver and kidney function in mice from the Ctrl, UTD, and SR CAR-M groups. **l** Organ weights from the Ctrl, UTD, and SR CAR-M groups were recorded. All values are expressed as mean ± SEM from *n* = 5 independent biological replicates, with each biological replicate performed in technical triplicate. *P < 0.05, **P < 0.01, and ***P < 0.001. *ALT* alanine transaminase, *AST* aspartate transaminase, *BUN* blood urea nitrogen, *Ctrl* control, *IFN* interferon, *ns* not significant, *PBS* phosphate-buffered saline, *SR* *CAR*-*M* switch receptor chimeric antigen receptor-macrophage, *TGF*-*β* transforming growth factor-beta, *UTD* untransduced.

Having demonstrated the multifaceted antitumor effects of SR CAR-M in vitro, we evaluated its therapeutic efficacy in an orthotopic 4T1 breast cancer model. We implanted 4T1-luciferase cells into the mammary fat pads of BALB/c mice and initiated treatment on day 7 with i.v. infusions of PBS, UTD-Ms, or SR CAR-Ms administered every 7 days (Fig. 5a). The presence of SR CAR-M in the organs was quantified by the in vivo imaging system. High DiR signal expression indicated infiltration and accumulation of SR CAR-M in the tumor, liver, spleen, and lung (Fig. 5b). Flow cytometric analysis of dissociated tumor tissues using anti-HA antibody staining confirmed sustained SR CAR-M persistence within tumors across days 7, 14, 21, and 28 post-treatments (Supplementary Fig. 10a). As expected, SR CAR-M treatment strongly inhibited tumor growth compared with the Ctrl and UTD groups (Fig. 5c–e).

To elucidate the mechanism underlying tumor suppression, we examined the remodeling of the immune microenvironment. Inflammatory cell infiltration in tumors was evaluated by histological analysis using hematoxylin and eosin (H&E) staining. As expected, the control and UTD groups showed low levels of inflammatory cell infiltration, whereas aggravated inflammatory cell infiltration was observed in the SR CAR-M group (Fig. 5f). Furthermore, macrophages isolated from SR CAR-M-treated tumors displayed markedly diminished expression of immunosuppressive (M2) markers IL-10, IL-4, and TGF-β and increased expression of pro-inflammatory (M1) markers, including TNF, IL-1, IL-6, IFNɣ, CXCL1, and CXCL2, compared with control tumor-isolated macrophages (Fig. 5g and Supplementary Fig. 11a). SR CAR-M treatment substantially reduced intratumoral IL-10 expression by days 14 and 28, whereas tumor IL-10 mRNA levels progressively increased in the control and UTD groups over 28 days, reflecting IL-10 consumption by SR CAR-M (Fig. 5h). Importantly, serum IL-10 levels remained unchanged across all groups at days 7, 14, and 21 (Supplementary Fig. 7a), indicating that local IL-10 consumption does not compromise systemic IL-10 homeostasis. Tissue-specific expression profiling demonstrated selective elevation of IFNγ, IL-1β, IL-6, and TNF in tumors from SR CAR-M-treated mice, with no corresponding increases in the lungs, spleen, or liver, indicating localized immune activation without systemic inflammation (Fig. 5I and Supplementary Fig. 11b). In addition, survival data for each group indicated that SR CAR-M-treated mice exhibited significantly prolonged survival compared with the control and UTD groups (Fig. 5j).

The safety of SR CAR-M treatment was also assessed. Serum liver enzymes (alanine transaminase and aspartate transaminase) and kidney function markers (blood urea nitrogen and creatinine) remained within the normal range (Fig. 5k) and organ weights remained comparable across all treatment groups (Fig. 5l). Extended monitoring (up to 75 days) confirmed the absence of organ damage, with normal organ weights (Supplementary Fig. 7b), unremarkable H&E histology (Supplementary Fig. 7c), and stable serum biochemistry (Supplementary Fig. 7d). Collectively, these findings establish that SR CAR-Ms selectively home to tumors reprogram the immune microenvironment by consuming IL-10 while inducing localized IFNγ production, suppress tumor growth, and prolong survival without systemic toxicity.

### SR CAR-Ms reprogram the tumor immune microenvironment and enhance antitumor immunity

Next, we examined the impact of SR CAR-M on the tumor immune landscape. SR CAR-M treatment fundamentally alters the phenotype of TAMs. We injected DiR-labeled UTD and SR CAR-M and isolated no DiR-labeled cells from the three groups using flow cytometry after 7 days. SR CAR-M treatment increased the macrophage population, characterized by elevated MHC II expression, a hallmark of antigen-presenting capacity (Supplementary Fig. 11c,d). The shift in macrophage phenotype was further evidenced by the increased expression of the M1 marker CD86 and the M2 marker CD206, with no change in PD-L1 expression (Supplementary Fig. 11e), suggesting complex TAM reprogramming.

**Fig. 6:**
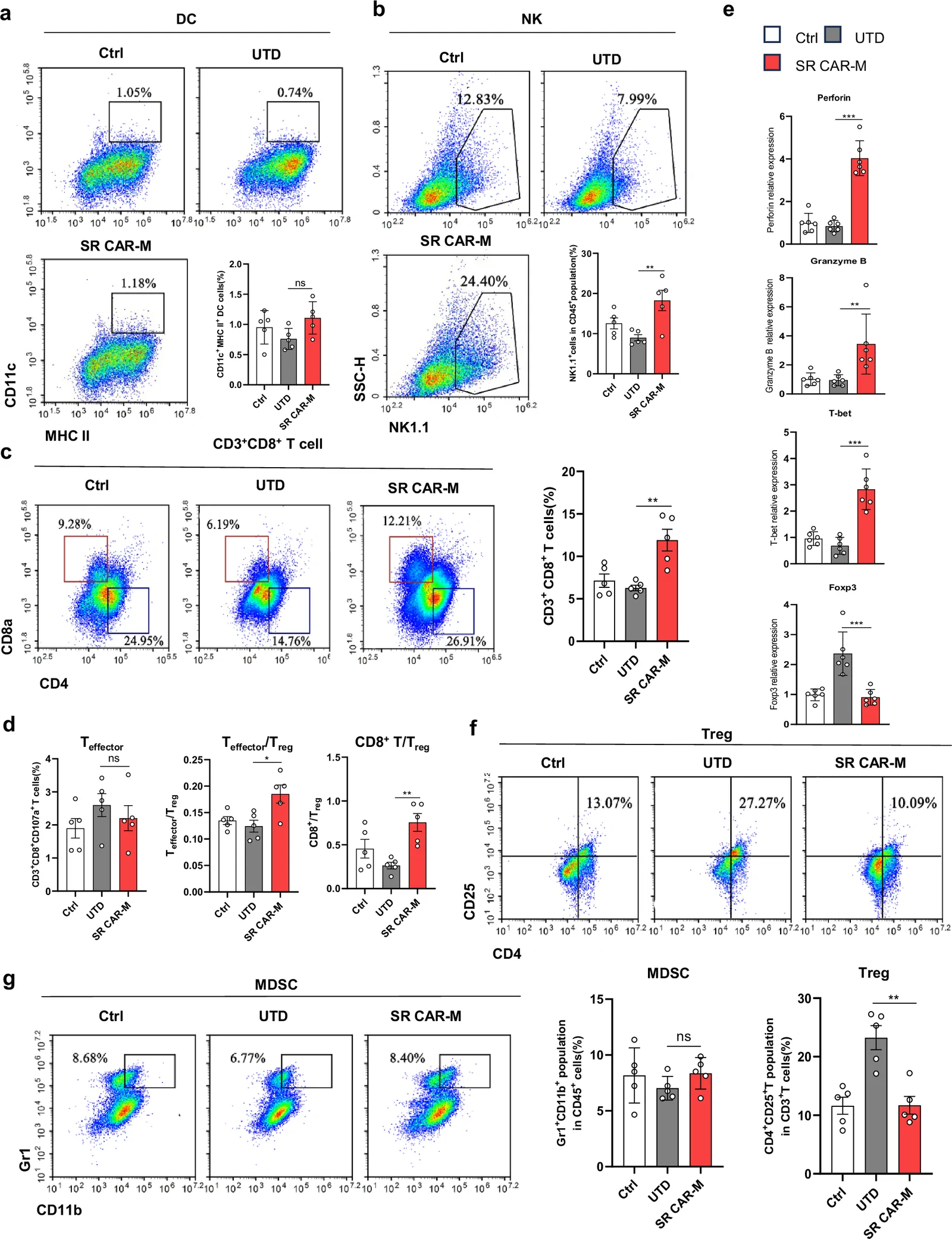
SR CAR-M improves TME in tumor-bearing mice. Single-cell suspensions were prepared from tumors, and flow cytometry was used to detect the infiltration of DCs (part **a**), NK cells (part **b**), and CD3^+^CD8^+^ T cells (part **c**) into the tumor microenvironment (TME) of 4T1 tumor-bearing mice treated with phosphate-buffered saline (PBS), UTD, or SR CAR-M. **d** Flow cytometry was used to detect the infiltration of T_eff_ cells in the TME of 4T1 tumor-bearing mice treated with PBS, UTD, and SR CAR-M and to analyze the CD8/T_reg_ and T_eff_/T_reg_ ratios. **e** The transcription levels of T-bet, Granzyme B, Perforin, and Foxp3 in tumor-bearing mice were detected using quantitative PCR. Flow cytometry was used to detect the infiltration of T_reg_ cells (part **f**) and MDSCs (part **g**) into the TME of 4T1 tumor-bearing mice treated with PBS, UTD, and SR CAR-M. All values are expressed as mean ± SEM from *n* = 5 independent biological replicates, with each biological replicate performed in technical triplicate. *P < 0.05, **P < 0.01, and ***P < 0.001. *Ctrl* control, *DC* dendritic cell, *MDSC* myeloid-derived suppressor cell, *MHC* major histocompatibility complex, *NK* natural killer, *ns* not significant, *SR* *CAR*-*M* switch receptor chimeric antigen receptor-macrophage, *UTD* untransduced.

This macrophage repolarization catalyzed a broader immune activation throughout the TME. SR CAR-M treatment enhanced NK cell recruitment and CD8^+^ T cell infiltration (Fig. 6b,c). Infiltrating T cells from SR CAR-M-treated mice exhibited markedly elevated expression of perforin, granzyme B, and the T_H_1 transcription factor T-bet, which was consistent with the increasing CD8^+^/T_reg_ and T_eff_/T_reg_ ratios in the SR CAR-M treatment group (Fig. 6d,e). Concomitantly, SR CAR-M treatment reduced the frequency of regulatory T cell populations, with the expression of Foxp3 being reduced in the infiltrating T cells from SR CAR-M-treated mice (Fig. 6e,f). By contrast, DCs and myeloid-derived suppressor cell infiltration remained unchanged (Fig. 6a,g). These findings demonstrate that SR CAR-Ms orchestrate comprehensive immune microenvironment remodeling, converting immunosuppressive tumor milieus into inflamed, T cell-enriched environments conducive to tumor rejection.

To confirm that these effects are attributable to signal switching rather than IL-10 sequestration or tonic TLR9 signaling, we evaluated the domain-deletion controls in vivo. In the orthotopic 4T1 model, only SR CAR-BM significantly enhanced DC, NK cell, and CD8⁺ T cell infiltration while reducing T_reg_ frequency and shifting TAMs toward an MHC II^high^ phenotype; neither ΔTLR9 SR CAR-M nor ΔIL-10R SR CAR-M achieved these effects (Supplementary Fig. 12a–h). Consistent with this immune remodeling, only SR CAR-BM suppressed tumor growth and reduced tumor weight (Supplementary Fig. 13a,b), with all groups maintaining favorable safety profiles (Supplementary Fig. 13c–f). These results definitively establish that the integrated signal-switching mechanism is essential for therapeutic efficacy.

### Armoring SR CAR-Ms with αPD-L1 enhances therapeutic efficacy

**Fig. 7:**
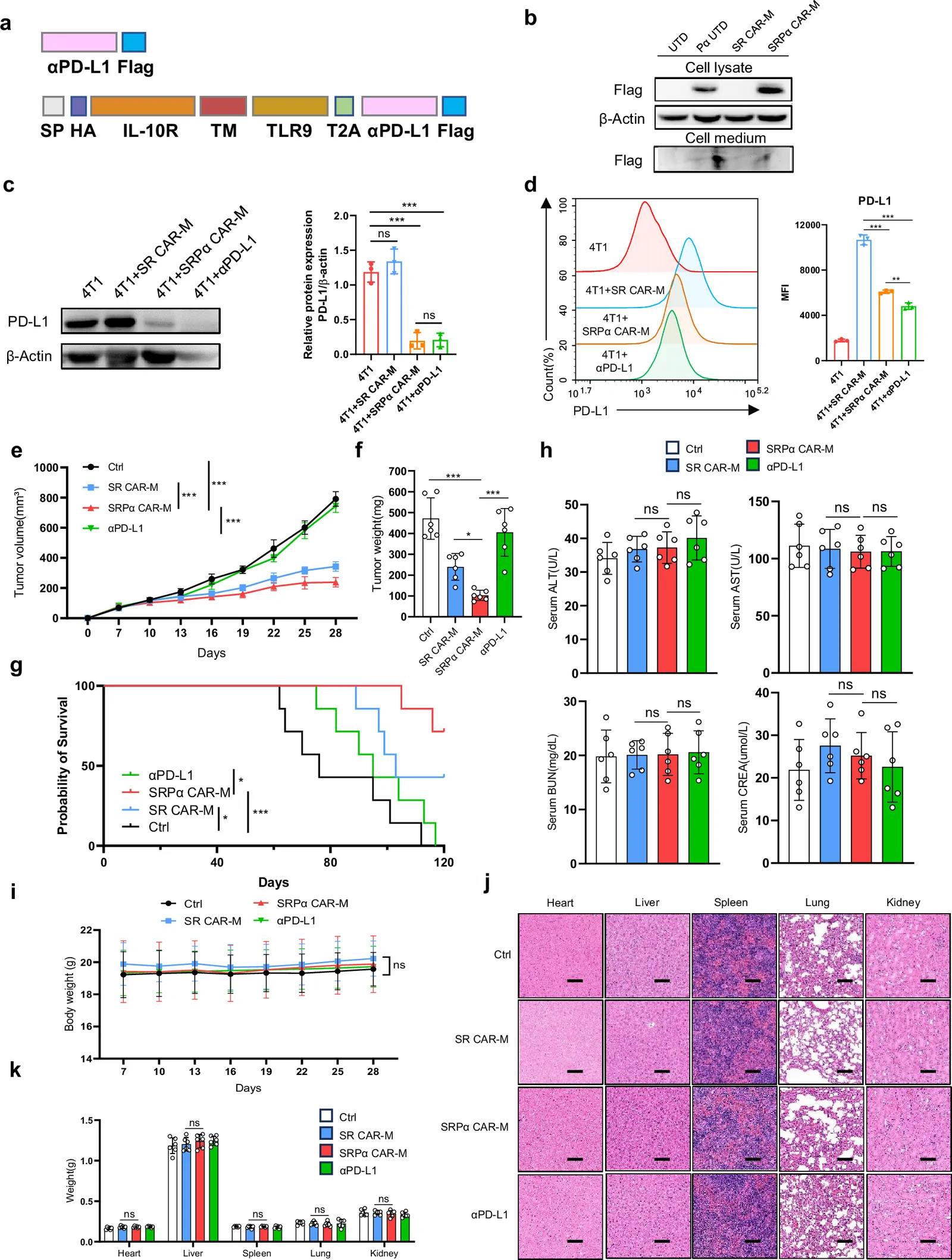
SR CAR-M armed with αPD-L1 enhances therapeutic efficacy in tumor-bearing mice. **a** Schematic representation of the SR CAR armed with αPD-L1 (SRPα CAR-M) construct design for macrophages. **b** Western blotting was used to detect the expression and secretion of αPD-L1 in UTD, UTD armed with αPD-L1 (Pα UTD), SR CAR-M, and SRPα CAR-M. Expression of PD-L1 in 4T1 cells after co-culture with SR CAR-M or SRPα CAR-M was determined by western blotting (part **c**) and flow cytometry (part **d**). αPD-L1 was used as a positive control. Tumor growth (part **e**) and tumor weight (part **f**) of mice in the control, SR CAR-M, SRPα CAR-M, and αPD-L1 groups were analyzed by measuring the tumor volume every 3 days. **g** Survival analysis of mice in the control, SR CAR-M, SRPα CAR-M, and αPD-L1 groups. **h** Analysis of serum biomarkers for liver and kidney function in mice from the control, SR CAR-M, SRPα CAR-M, and αPD-L1 groups. **i** Body weights of mice in each group. Representative hematoxylin and eosin images of normal organs from mice with the indicated treatments (part **j**) and organ weights were recorded (part **k**). All values are expressed as mean ± SEM from *n* = 6 independent biological replicates, with each biological replicate performed in technical triplicate (except parts **b**–**d**, which is *n* = 3). *P < 0.05, **P < 0.01, and ***P < 0.001. *ALT* alanine transaminase, *AST* aspartate transaminase, *BUN* blood urea nitrogen, *Ctrl* control, *MFI* mean fluorescence intensity, *ns* not significant, *SR* *CAR*-*M* switch receptor chimeric antigen receptor-macrophage, *TLR* Toll-like receptor, *UTD* untransduced.

To further enhance therapeutic efficacy, we engineered SR CAR-M to secrete anti-PD-L1 antibodies, reasoning that a localized checkpoint blockade might synergize with signal-mediated immune activation. We engineered a dual-function construct that incorporated both the SR CAR and a secretable anti-PD-L1 single-chain variable fragment (αPD-L1), creating what we termed SRPα CAR-M (Fig. 7a). SRPα CAR-M and Pα UTD (macrophages only armed with αPD-L1 construct) efficiently expressed and secreted functional anti-PD-L1 antibodies, as evidenced by their detection in both cellular lysates and culture supernatants (Fig. 7b). To validate that the secreted antibody retained its checkpoint-blocking function, we co-cultured 4T1 tumor cells with SR CAR-M and SRPα CAR-M. As expected, SRPα CAR-Ms substantially reduced PD-L1 protein levels on tumor cell surfaces, achieving a reduction comparable to that observed with direct anti-PD-L1 antibody treatment (Fig. 7c,d). This finding confirmed that SRPα CAR-Ms effectively neutralized PD-L1 in the TME.

Next, we evaluated whether this dual-targeting strategy improved the therapeutic efficacy in tumor-bearing mice. The combination approach was highly effective. SRPα CAR-M treatment achieved even greater suppression, limiting tumor growth compared with SR CAR-M (Fig. 7e,f). Importantly, this dual approach markedly outperformed anti-PD-L1 monotherapies. The superior tumor control achieved by SRPα CAR-Ms translated directly to improved survival outcomes, with treated mice demonstrating extended median survival compared with those treated with SR CAR-M alone, anti-PD-L1 alone, and control groups (Fig. 7g).

Finally, the safety of SRPα CAR-Ms treatment was assessed. Reassuringly, serum biomarkers for liver function (alanine transaminase and aspartate transaminase) and kidney function (blood urea nitrogen and creatinine) remained within the normal range across all treatment groups, including mice receiving SRPα CAR-Ms (Fig. 7h). This finding demonstrated that the combination strategy maintained the favorable safety profile previously observed with SR CAR-Ms alone. More importantly, there was no obvious weight loss in the SRPα CAR-Ms group throughout the treatment, indicating a low potential for systemic toxicity (Fig. 7i). Finally, the toxicities related to the five major organs were evaluated by histological analysis using H&E staining. As expected, there was no observed serious damage in H&E-stained sections of the heart, liver, spleen, lung, and kidney (Fig. 7j). In addition, no significant differences in organ weights were observed among the four groups. Collectively, these data revealed that arming SRPα CAR-Ms reduced tumor burden without compromising safety of the host.

## Discussion

The TME orchestrates immunosuppression through multiple mechanisms, among which IL-10 stands as a particularly influential mediator. Elevated levels of across diverse malignancies correlate with adverse clinical outcomes. IL-10 actively suppresses antitumor immunity by inhibiting DC maturation, promoting regulatory T cell differentiation, and polarizing macrophages toward tumor-supportive M2 phenotypes^30^. Although systemic IL-10 blockade represents an intuitive therapeutic strategy, clinical translation has proven challenging because of IL-10’s complex, context-dependent immunoregulatory functions^31^. Here, we developed an alternative paradigm: instead of globally neutralizing IL-10, we engineered macrophages to exploit this immunosuppressive signal as a location-specific trigger for immune activation. Our SR CAR-M platform embodies a signal-switching strategy, wherein the IL-10Rα extracellular domain captures IL-10 and channels this input through TLR9 intracellular signaling machinery, effectively converting an immunosuppressive stimulus into pro-inflammatory activation. This design offers several conceptual advantages. First, SR CAR-Ms function as conditional therapeutics, selectively activating in IL-10-rich TMEs while remaining quiescent in healthy tissues. This spatial specificity mitigates concerns regarding systemic immune dysregulation accompanying broad IL-10 neutralization^32^. Second, tumor cells themselves provide the activating signal by secreting IL-10 concentrations approaching 2000 pg/ml in the case of 4T1 breast cancer, thereby transforming a tumor defense mechanism into a therapeutic vulnerability. Third, SR CAR-Ms simultaneously deplete local IL-10 while secreting pro-inflammatory mediators, creating a dual mechanism for microenvironment remodeling. Our domain-deletion experiments confirm that this dual mechanism is inseparable: ΔTLR9 SR CAR-M, which sequesters IL-10 without generating a pro-inflammatory signal, failed to replicate the therapeutic effects of the full-length construct either in vitro or in vivo (Supplementary Figs. 3, 8, 12, and 13), demonstrating that IL-10 consumption alone is insufficient and must be coupled with active signal inversion.

A central mechanistic question is how the TLR9 intracellular domain initiates signaling at the cell surface, given that canonical TLR9 activation requires endosomal trafficking and CpG DNA-induced dimerization^33^. Structural studies have established that TIR domain dimerization is sufficient for MyD88 recruitment independently of subcellular compartmentalization^34^. Two lines of experimental evidence define the signaling mode of our chimeric receptor. First, siRNA-mediated MyD88 knockdown abolished all downstream signaling outputs, including NF-κB nuclear translocation, cytokine secretion, and pro-inflammatory gene transcription, establishing that the SR CAR is fully dependent on canonical TLR9 adaptor machinery. Second, chloroquine pretreatment, which blocks endosomal acidification and is sufficient to abrogate canonical TLR9 signaling, had no detectable effect on SR CAR-mediated NF-κB activation or pro-inflammatory gene expression. Together, these results demonstrate that the SR CAR operates through what we term a “synthetic activation, canonical signaling” paradigm: the upstream trigger is non-canonical and endosome-independent, but the downstream signaling architecture recapitulates canonical TLR9 outputs. We propose that IL-10 binding induces receptor oligomerization at the plasma membrane, bringing TLR9 intracellular domains into proximity for MyD88 recruitment, analogous to how CARs co-opt CD3ζ signaling through ligand-induced clustering^35^. Structural studies have established that TIR domain dimerization is sufficient for MyD88 recruitment independently of subcellular compartmentalization, providing a mechanistic basis for this model. Direct demonstration of IL-10-induced receptor oligomerization, for example, via proximity ligation assay or co-immunoprecipitation, remains an important direction for future investigation. TLR9 was selected over other MyD88-dependent TLRs for three reasons: it activates both NF-κB and IRF pathways, providing dual pro-inflammatory output; unlike TLR4, it signals exclusively through MyD88 without requiring TRIF^36^, and TLR9 agonism has been extensively shown to promote M1 polarization and antitumor immunity^37^.

The therapeutic landscape for solid tumors has proven notably resistant to adoptive cellular immunotherapies, which have revolutionized the treatment of hematologic malignancies. Despite the transformative success of CAR-T cell therapy in B cell lymphomas, formidable obstacles are encountered in solid tumors, including inadequate infiltration, immunosuppressive microenvironments, and limited persistence^38,39^. Recent advances in CAR-M technology have demonstrated antitumor efficacy through tumor antigen recognition and enhanced phagocytosis^10^. However, these approaches primarily target tumor cells directly via surface antigens, making them susceptible to immune escape through antigen loss or heterogeneity, which are well-documented resistance mechanisms^8^. Our SR CAR-M strategy diverges fundamentally by targeting the immunosuppressive architecture^39^. By reprogramming the microenvironment rather than directly killing tumor cells, SR CAR-Ms potentiate endogenous antitumor immunity by recruiting and activating NK cells, DCs, and cytotoxic T lymphocytes. This indirect mechanism may be less vulnerable to tumor evolution, as IL-10 production represents a core immunosuppressive strategy that is broadly conserved across tumor types.

A particularly compelling aspect of SR CAR-Ms is their resistance to microenvironmental reprogramming. Conventional M1-polarized macrophages rapidly revert to immunosuppressive phenotypes upon tumor infiltration, a phenomenon that has limited macrophage therapy approaches^10^. Our data demonstrate that SR CAR-Ms maintain M1 characteristics even when challenged with IL-4, IL-13, and TCM, which potently induce M2 polarization in control macrophages. Critically, direct comparison with LPS/IFNγ-polarized M1 macrophages revealed that conventionally polarized cells rapidly reverted upon TCM exposure, whereas SR CAR-Ms maintained their phenotype. This distinction underscores a fundamental advantage of the signal-switching architecture: the continuous presence of IL-10 in the TME, which normally drives immunosuppression, instead actively reinforces M1 polarization through sustained TLR9 activation, creating a self-sustaining pro-inflammatory feedback loop that is resistant to microenvironmental reprogramming^40^. This phenotypic stability derives from active signal switching, as tumor-derived IL-10 continuously reinforces TLR9 pathway activation, creating a self-sustaining pro-inflammatory state. Importantly, SR CAR-M.IL-10 not only maintained its own M1 phenotype but also deactivated neighboring M2 macrophages through paracrine mechanisms, suggesting the potential for TAM repolarization beyond the initially infused cells.

The orchestration of adaptive immunity by SR CAR-Ms represents an additional layer of therapeutic activity. By rescuing DC maturation from IL-10 suppression and directly activating T cells, SR CAR-Ms establish conditions for sustained antitumor responses. The prevention of T cell exhaustion, as evidenced by the suppressed expression of PD-1, LAG-3, Tim-3, TIGIT, and CTLA-4, is a major barrier to cancer immunotherapy^41^. A balanced consideration of IL-10 biology is warranted here, as IL-10 has been shown to support CD8⁺ T cell survival and function under certain conditions^42,43^, consistent with our own observation that IL-10 alone enhances CD8⁺ T cell proliferation. However, our in vivo data demonstrate that the net immunological effect of IL-10 consumption by SR CAR-Ms is overwhelmingly pro-inflammatory and antitumorigenic, as evidenced by enhanced CD8⁺ T cell infiltration, elevated effector molecule expression, and reduced exhaustion markers. We speculate that the concurrent secretion of IFNγ, IL-6, and TNF-α by activated SR CAR-Ms, together with rescued DC function, functionally compensates for any loss of direct IL-10-mediated T cell support. This observation motivated our strategy of combining the armoring of SR CAR-Ms with secretable anti-PD-L1 antibodies^44^.

The resulting SRPα CAR-Ms achieved superior tumor control compared with either monotherapy, validating the concept that macrophages can serve as vehicles for the localized delivery of checkpoint blockade. This approach has broad applicability to multiple solid tumors, especially immunologically ‘cold’ cancers, and cellular delivery holds potential across not only CAR-T cells but also CAR-engineered NK cells, macrophages, and non-conventional T cells, in disease settings that warrant immune checkpoint blockade and cytokine delivery to improve therapeutic responses. This approach may circumvent the limitations of systemic checkpoint inhibitor administration, including immune-related adverse events and limited intratumoral drug penetration^20^.

This study has several limitations. First, although our murine models provide proof of concept, immunological differences between species necessitate rigorous validation in human systems before clinical translation. Our preliminary human macrophage data and primary BMDM validation are encouraging, but comprehensive studies in humanized mouse models remain essential. Encouragingly, the first-in-human phase I trial of CT-0508 CAR-M targeting HER2 demonstrated feasibility and safety, establishing the viability of CAR-M therapy^11^. Second, the biodistribution data revealed SR CAR-M accumulation in the liver and spleen alongside tumors, raising questions about enhancing tumor-specific trafficking. Although localized immune activation was observed selectively in tumors, reassuringly, serum IL-10 levels remained unchanged across all treatment groups throughout the observation period, suggesting that SR CAR-M-mediated IL-10 consumption is largely confined to the TME and does not perturb systemic IL-10 homeostasis^45^. Nevertheless, strategies to further enrich tumor homing, such as chemokine receptor engineering or local administration, merit investigation. Third, the optimal dosing regimen, treatment schedule, and potential synergies with radiotherapy, chemotherapy, and other immunotherapies require systematic exploration. Fourth, the biphasic dose–response at supraphysiological IL-10 concentrations (Fig. 1g), likely reflecting engagement of endogenous IL-10R complexes, warrants attention for tumors with exceptionally high IL-10 levels; receptor engineering to increase binding affinity or reduce endogenous IL-10R expression could address this in future iterations.

Looking forward, the signal-switching platform extends beyond IL-10 targeting in cancer therapy. Other immunosuppressive factors enriched in tumors, including TGF-β, prostaglandin E2, and adenosine, could theoretically serve as activation signals using analogous receptor engineering approaches^46^. The modular nature of our design, demonstrated by the successful incorporation of anti-PD-L1 secretion, suggests the possibility of multifunctional therapeutic platforms tailored to specific TMEs. Furthermore, SR CAR technology may find applications in inflammatory diseases in which localized immune modulation is desired, essentially inverting our approach by converting pro-inflammatory signals into tissue-protective responses.

In conclusion, SR CAR-Ms establish signal switching as a powerful strategy for cellular immunotherapy, transforming inhibitory signals into therapeutic opportunities. By simultaneously consuming IL-10, activating innate and adaptive immunity, and delivering localized checkpoint blockade, this platform addresses multiple immunosuppressive mechanisms within tumors, offering new possibilities for treating solid malignancies that are resistant to conventional immunotherapies^47^.

In conclusion, we established signal switching as a powerful strategy for tumor-selective macrophage immunotherapy by transforming IL-10, the most abundant immunosuppressive cytokine in solid tumors, into a location-specific activation trigger through MyD88-dependent TLR9 intracellular signaling. SR CAR-Ms simultaneously deplete local IL-10, activate pro-inflammatory responses within tumors, and maintain phenotypic stability that surpasses conventional LPS/IFNγ-driven M1 polarization, despite persistent immunosuppressive pressure. Domain-deletion controls confirm that both IL-10 binding and TLR9 signaling are indispensable for this phenotype, excluding IL-10 sequestration or tonic signaling as alternative explanations. Unlike conventional CAR-M approaches targeting tumor antigens, SR CAR-M addresses the immunosuppressive architecture itself, converting a core tumor defense mechanism into therapeutic vulnerability. The dual-functional SRPα CAR-M platform demonstrated that macrophages can deliver a localized checkpoint blockade while remodeling the TME. Validation in primary murine bone marrow-derived macrophages and human PBMC-derived macrophages confirms cross-species conservation of the signal-switching mechanism and supports translational applicability. Beyond IL-10, this signal-switching paradigm extends to other TME-enriched immunosuppressive factors, enabling the development of customizable platforms tailored to specific tumor contexts. As cellular immunotherapy evolves from direct tumor targeting to microenvironment reprogramming, SR CAR-M represents a versatile approach that addresses multiple immunosuppressive mechanisms simultaneously, offering new possibilities for treating solid malignancies resistant to conventional immunotherapies.

## Supplementary information

## Data Availability

All data are available from the corresponding author.
