# Supplementary material for: Engineered macrophages with IL-10–TLR9 signal switch receptors for reprogramming tumor microenvironment and enhancing antitumor immunity
Source: Exp Mol Med. 2026 Aug 6;58(8):2701–18. doi: 10.1038/s12276-026-01800-5 (PMC13538573; doi:10.1038/s12276-026-01800-5)
Supplement: Supplementary file 1 [file 12276_2026_1800_MOESM1_ESM.pdf]

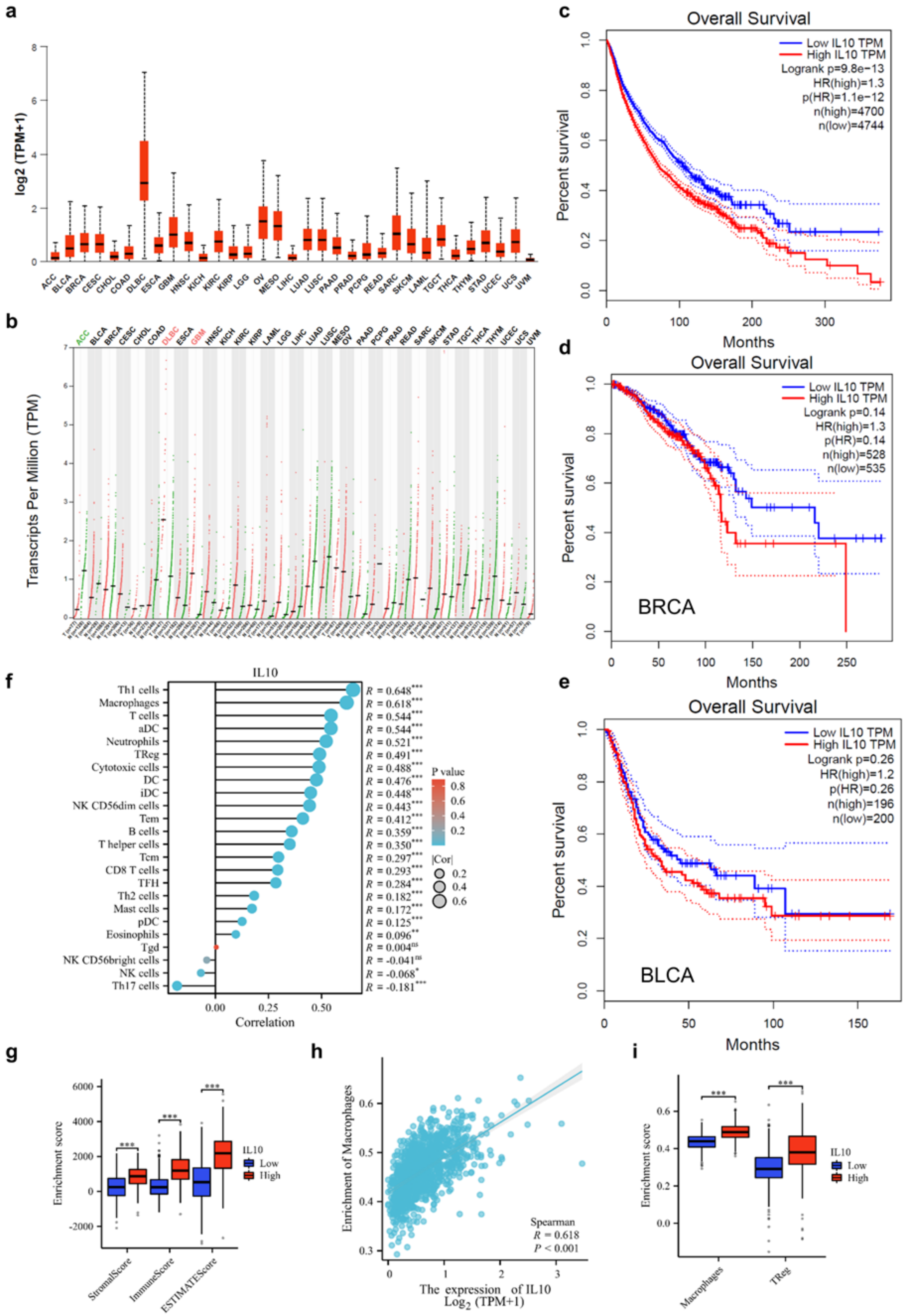

a
log2 (TPM+1)
ACC BLCA BRCA CESC CHOL COAD DLBC ESCA GBM HNSC KICH KIRC KIRP LGG OV MESO LIHC LUAD LUSC PAAD PRAD PCPG READ SARC SKCM LAML TGCT THCA THYM STAD UCEC UCS UVM
b
Transcripts Per Million (TPM)
ACC BLCA BRCA CESC CHOL COAD DLBC ESCA GBM HNSC KICH KIRC KIRP LAML LGG LIHC LUAD LUSC MESO OV PAAD PCPG PRAD READ SARC SKCM STAD TGCT THCA THYM UCEC UCS UVM
c
Overall Survival
Low IL10 TPM
High IL10 TPM
Logrank p=9.8e−13
HR(high)=1.3
p(HR)=1.1e−12
n(high)=4700
n(low)=4744
Percent survival
Months
d
Overall Survival
Low IL10 TPM
High IL10 TPM
Logrank p=0.14
HR(high)=1.3
p(HR)=0.14
n(high)=528
n(low)=535
BRCA
Percent survival
Months
e
Overall Survival
Low IL10 TPM
High IL10 TPM
Logrank p=0.26
HR(high)=1.2
p(HR)=0.26
n(high)=196
n(low)=200
BLCA
Percent survival
Months
f
IL10
Th1 cells R = 0.648***
Macrophages R = 0.618***
T cells R = 0.544***
aDC R = 0.544***
Neutrophils R = 0.521***
TReg R = 0.491***
Cytotoxic cells R = 0.488***
DC R = 0.476***
iDC R = 0.448***
NK CD56dim cells R = 0.443***
Tem R = 0.412***
B cells R = 0.359***
T helper cells R = 0.350***
Tcm R = 0.297***
CD8 T cells R = 0.293***
TFH R = 0.284***
Th2 cells R = 0.182***
Mast cells R = 0.172***
pDC R = 0.125***
Eosinophils R = 0.096**
Tgd R = 0.004ns
NK CD56bright cells R = -0.041ns
NK cells R = -0.068*
Th17 cells R = -0.181***
P value
|Cor|
Correlation
g
Enrichment score
IL10
Low
High
StromalScore
ImmuneScore
ESTIMATEScore
h
Enrichment of Macrophages
Spearman
R = 0.618
P < 0.001
The expression of IL10
Log2 (TPM+1)
i
Enrichment score
IL10
Low
High
Macrophages
TReg

Supplementary Fig. 1: The Role Of IL-10 In TME

a. IL10 mRNA expression in cancer samples from TCGA dataset. **b**. Comparison of the expression of IL10 in cancer samples with that in normal samples from the TCGA and GTEx datasets. **c**. Kaplan–Meier analysis of overall survival (OS) in patients with various types of cancers based on IL10 expression, including ACC, BLCA, BRCA, CESC, CHOL, COAD, DLBC, ESCA, GBM, HNSC, KICH, KIRC, KIRP, LAML, LGG, LIHC, LUAD, LUSC, MESO, OV, PAAD, PCPG, PRAD, READ, SARC, SKCM, STAD, TGCT, THCA, THYM, UCEC, UCS, and UVM. **d-e**. Patients with (**d**) BRCA and (**e**) BLCA were individually analyzed using Kaplan–Meier survival analysis. The p-value was generated using the log-rank test. **f**. The relationship between IL10 expression and immune cell infiltration was analyzed using single-sample GSEA (ssGSEA) in BRCA. The Wilcoxon rank-sum test was used to analyze significant differences. **g**. Relationship between IL10 expression and estimated scores, immune scores, and stromal scores in BRCA. **h**. Relationship between IL10 expression and Macrophages in BRCA. p-value was generated using the Spearman test. **i**. Relationship between IL10 expression and Macrophages and Treg cells in BRCA.

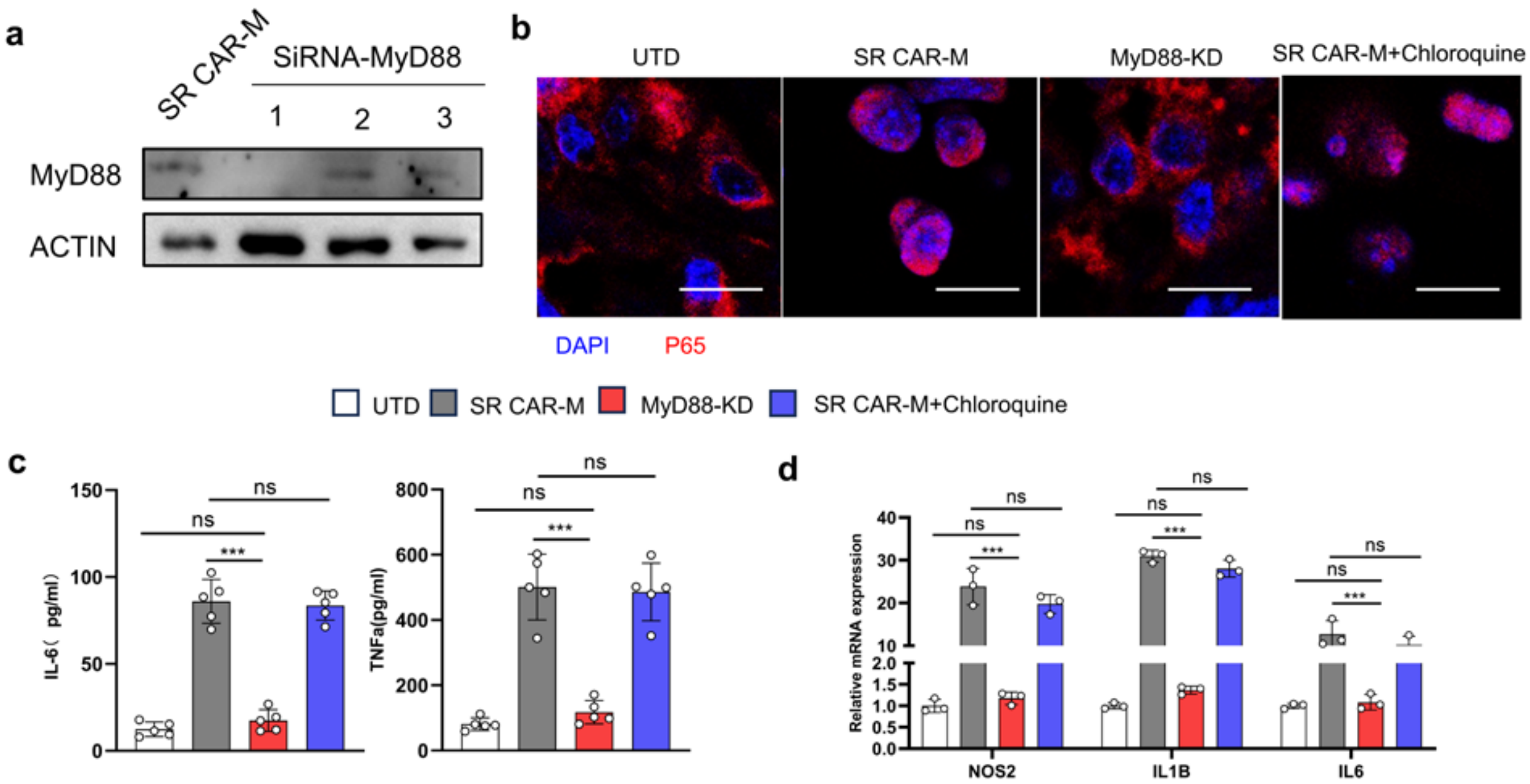

Supplementary Fig. 2: MyD88-dependent, endosome-independent signaling underlies the SR CAR signal switch.

a. Western blot analysis of MyD88 expression following siRNA-mediated knockdown in SR CAR-M cells. **b**. Immunofluorescence analysis of NF-κB p65 intracellular distribution in UTD, SR CAR-M, MyD88-KD SR CAR-M, and chloroquine-pretreated SR CAR-M (50 μM, 1 h pretreatment) after IL-10 stimulation (100 ng/mL, 6 h). Scale bars, XX μm. **c**. ELISA quantification of IL-6 and TNF-α secretion by UTD, SR CAR-M, and MyD88-KD SR CAR-M following IL-10 stimulation. **d**. qPCR analysis of NOS2, IL-1β, and IL-6 transcription in UTD, SR CAR-M, MyD88-KD SR CAR-M, and chloroquine-pretreated SR CAR-M after IL-10 stimulation (100 ng/mL, 24 h). All values are expressed as mean ± SEM from n = 3 independent biological replicates, with each biological replicate performed in technical triplicate (Except **c** which is n=5). *P < 0.05, **P < 0.01, and ***P < 0.001.

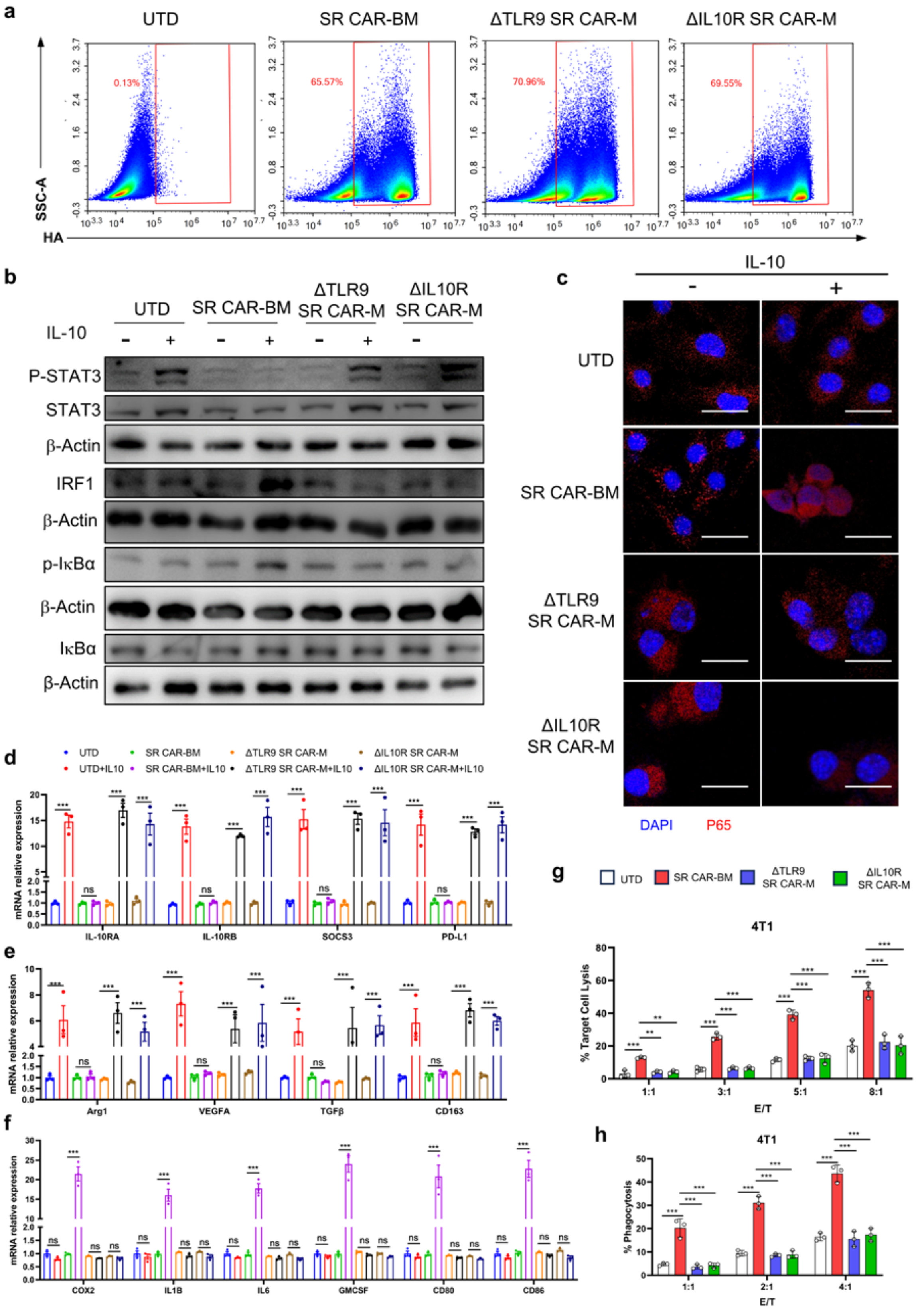

a
UTD
SR CAR-BM
ΔTLR9 SR CAR-M
ΔIL10R SR CAR-M
0.13%
65.57%
70.96%
69.55%
SSC-A
HA
b
IL-10
P-STAT3
STAT3
β-Actin
IRF1
p-IκBα
IκBα
c
IL-10
DAPI
P65
d
UTD
SR CAR-BM
ΔTLR9 SR CAR-M
ΔIL10R SR CAR-M
UTD+IL10
SR CAR-BM+IL10
ΔTLR9 SR CAR-M+IL10
ΔIL10R SR CAR-M+IL10
mRNA relative expression
IL-10RA
IL-10RB
SOCS3
PD-L1
e
Arg1
VEGFA
TGFβ
CD163
f
COX2
IL1B
IL6
GMCSF
CD80
CD86
g
4T1
% Target Cell Lysis
E/T
1:1
3:1
5:1
8:1
h
% Phagocytosis
2:1
4:1

Supplementary Fig. 3: Generation and characterization of SR CAR-BM, ΔTLR9 SR CAR-M and ΔIL10R SR CAR-M

a. Representative flow cytometric analysis of transduction efficiency (HA-tag expression) on BMDM 2 days after transduction with SR CAR, ΔTLR9 SR CAR and ΔIL10R SR CAR. **b**. The TLR9 signalling pathway IKBα and p-IKBα and IL10 signalling pathway STAT3, p-STAT3 and IRF1 on SR CAR-BM, ΔTLR9 SR CAR-M, ΔIL10R SR CAR-M or UTD in response to IL-10 stimulation were detected by western blot analysis. **c**. The intracellular distribution of the NFκB subunit P65 in SR CAR-Mac cells after stimulation with IL-10 (100 ng/mL) for 6 h was detected by immunofluorescence. **d**. The mRNA expression of IL10RA,IL10RB,SOCS3 and PD-L1 of IL10 signalling pathway was examined by qPCR. **e-f**. Transcription levels of (**e**) anti-inflammatory(M2) genes and (**f**) pro-inflammatory (M1) genes on SR CAR-BM, ΔTLR9 SR CAR-M, ΔIL10R SR CAR-M or UTD in response to IL-10 were detected by qPCR after IL-10 (100 ng/mL) stimulation for 1 day. **g**. Killing assay of 4T1 cells by SR CAR-BM, ΔTLR9 SR CAR-M, ΔIL10R SR CAR-M or UTD cells at different E: T ratios (Macrophage: Tumor = 1:1, 3:1, 5:1, 8:1). **h**. The phagocytosis of SR CAR-BM, ΔTLR9 SR CAR-M, ΔIL10R SR CAR-M or UTD against 4T1 cells at different E: T ratios. All values are expressed as mean ± SEM from n = 3 independent biological replicates, with each biological replicate performed in technical triplicate. *P < 0.05, **P < 0.01,and ***P < 0.001.

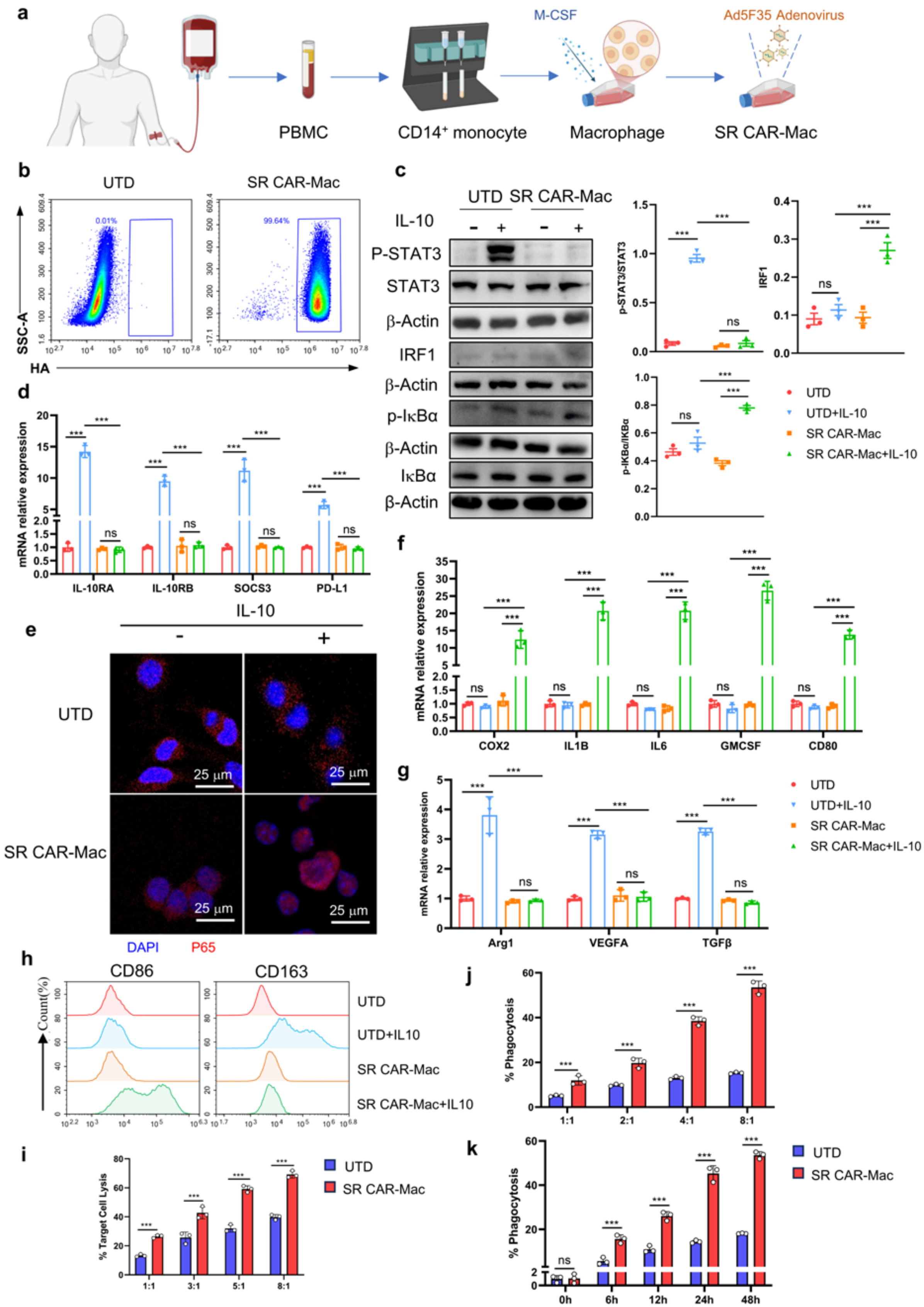

a
M-CSF
Ad5F35 Adenovirus
PBMC
CD14⁺ monocyte
Macrophage
SR CAR-Mac
b
UTD
SR CAR-Mac
0.01%
99.64%
SSC-A
HA
c
UTD SR CAR-Mac
IL-10
P-STAT3
STAT3
β-Actin
IRF1
β-Actin
p-IκBα
β-Actin
IκBα
β-Actin
p-STAT3/STAT3
IRF1
p-IKBα/IKBα
UTD
UTD+IL-10
SR CAR-Mac
SR CAR-Mac+IL-10
d
mRNA relative expression
IL-10RA
IL-10RB
SOCS3
PD-L1
e
IL-10
UTD
SR CAR-Mac
25 μm
DAPI
P65
f
mRNA relative expression
COX2
IL1B
IL6
GMCSF
CD80
g
mRNA relative expression
Arg1
VEGFA
TGFβ
UTD
UTD+IL-10
SR CAR-Mac
SR CAR-Mac+IL-10
h
CD86
CD163
Count(%)
UTD
UTD+IL10
SR CAR-Mac
SR CAR-Mac+IL10
i
% Target Cell Lysis
1:1
3:1
5:1
8:1
UTD
SR CAR-Mac
j
% Phagocytosis
1:1
2:1
4:1
8:1
k
% Phagocytosis
0h
6h
12h
24h
48h
UTD
SR CAR-Mac

Supplementary Fig. 4: Generation and characterization of SR CAR-Mac

a. Schematic representation of the preparation process of SR CAR-Mac. **b**. Representative flow cytometric analysis of transduction efficiency (HA-tag expression) on macrophages(PBMC-derived) 2 days after transduction with SR CAR. **c**. The TLR9 signalling pathway IKBα and p-IKBα and IL10 signalling pathway STAT3, p-STAT3 and IRF1 on SR CAR-Mac or UTD in response to IL-10 stimulation were detected by western blot analysis, and their gray-scale statistical analysis was shown (right). **d**. The mRNA expression of IL10RA,IL10RB,SOCS3 and PD-L1 of IL10 signalling pathway was examined by qPCR. **e**. The intracellular distribution of the NFκB subunit P65 in SR CAR-Mac cells after stimulation with IL-10 (100 ng/mL) for 6 h was detected by immunofluorescence. **f-g**. Transcription levels of (**f**) pro-inflammatory (M1) genes and (**g**) anti-inflammatory(M2) genes on SR CAR-Mac or UTD in response to IL-10 were detected by qPCR after IL-10 (100 ng/mL) stimulation for 1 day. **h**. Representative flow cytometric analysis of M1(CD86) and M2(CD163) markers. **i**. Killing assay of breast cancer cells(MDA-MB-231) by UTD and SR CAR-Mac cells at different E: T ratios (Macrophage: Tumor = 1:1, 3:1, 5:1, 8:1). **j-k**. The phagocytosis of UTD and SR CAR-Mac against breast cancer cells(MDA-MB-231) at different (**j**) E: T ratios and (**k**) time points. All values are expressed as mean ± SEM from n = 3 independent biological replicates, with each biological replicate performed in technical triplicate. *P < 0.05, **P < 0.01,and ***P < 0.001.

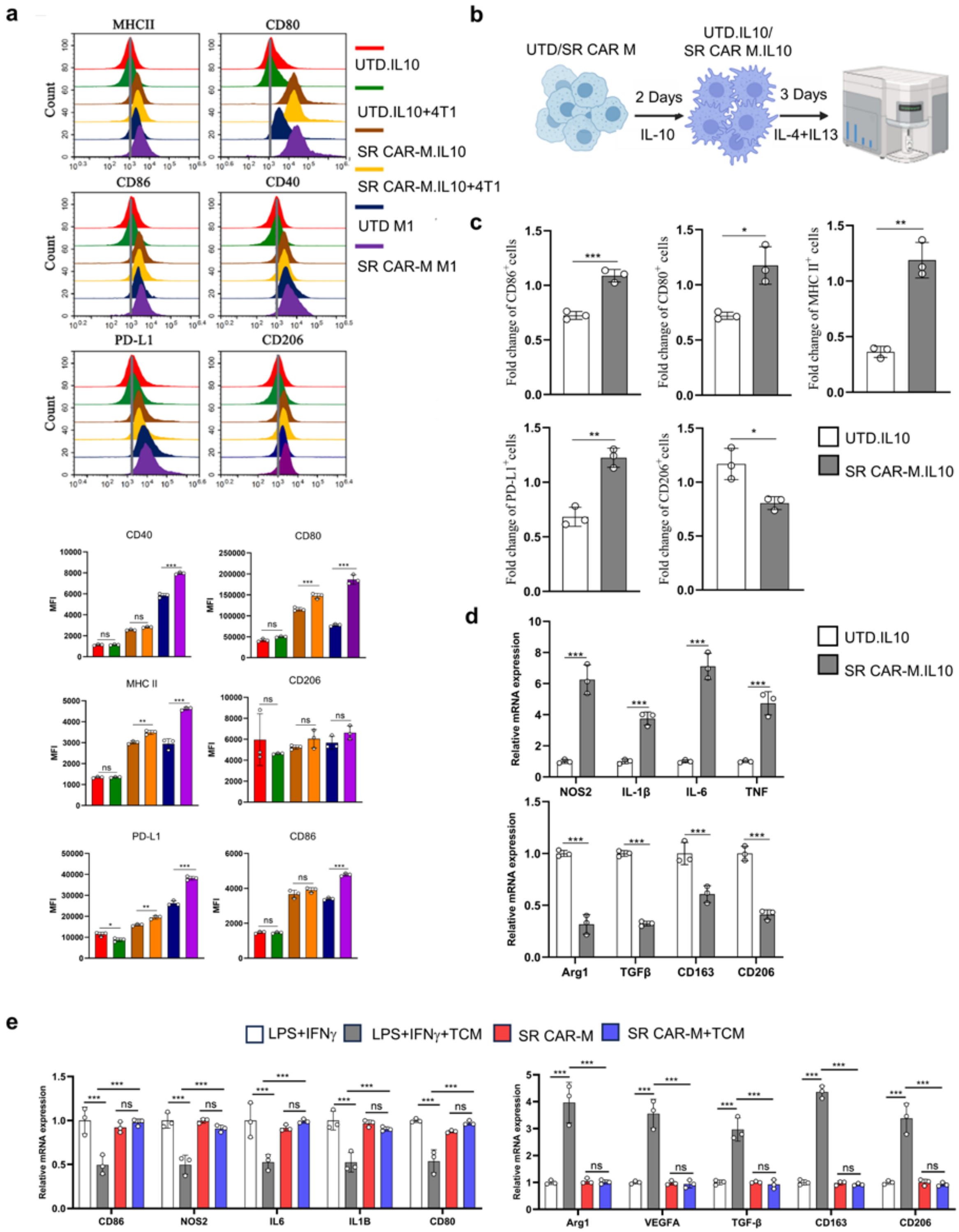

a
MHCII
CD80
CD86
CD40
PD-L1
CD206
Count
UTD.IL10
UTD.IL10+4T1
SR CAR-M.IL10
SR CAR-M.IL10+4T1
UTD M1
SR CAR-M M1
MFI
b
UTD/SR CAR M
2 Days
IL-10
UTD.IL10/
SR CAR M.IL10
3 Days
IL-4+IL13
c
Fold change of CD86+ cells
Fold change of CD80+ cells
Fold change of MHC II+ cells
Fold change of PD-L1+ cells
Fold change of CD206+ cells
UTD.IL10
SR CAR-M.IL10
d
Relative mRNA expression
NOS2
IL-1β
IL-6
TNF
Arg1
TGFβ
CD163
CD206
e
LPS+IFNγ
LPS+IFNγ+TCM
SR CAR-M
SR CAR-M+TCM
CD86
NOS2
IL6
IL1B
CD80
Arg1
VEGFA
TGF-β
CD163
CD206

Supplementary Fig. 5: **SR CAR-Ms Were Resistant to M2-Inducing Cytokines in Presence of IL-10**

a. We stimulated UTD and SR CAR-M with LPS (100 ng/mL)/IFN-g (25 ng/mL) or IL-10 (100 ng/mL) for two days. Then, UTD.IL10 and SR CAR-M cells were added. IL10 were co-cultured with 4T1 cells for 24 h and isolated using F4/80 beads. Flow cytometry analysis was performed to detect M1/M2 phenotype-related markers on the membrane surface (CD80, CD86, MHC II, CD40, PD-L1, and CD206). **b**. Experimental schedule for UTD and SR CAR-M stimulation. **c**. Flow cytometry analysis was performed to detect M1/M2 phenotype-related markers (CD80, CD86, MHC II, PD-L1, and CD206) on the membrane surface of UTD.IL10 and SR CAR-M cells. IL10 in the presence of M2-inducing cytokines. **d**. M1/M2 phenotype-related gene expression of UTD.IL10 and SR CAR-M.IL10 expression after M2-inducing cytokine stimulation was examined using qPCR. **e**. Transcription levels of pro-inflammatory (M1) genes (left) and anti-inflammatory(M2) genes (right) on SR CAR-M or M1 macrophage in response to IL-10 were detected by qPCR after IL-10 (100 ng/mL) stimulation for 1 day. All values are expressed as mean ± SEM from n = 3 independent biological replicates, with each biological replicate performed in technical triplicate. *P < 0.05, **P < 0.01, and ***P < 0.001.

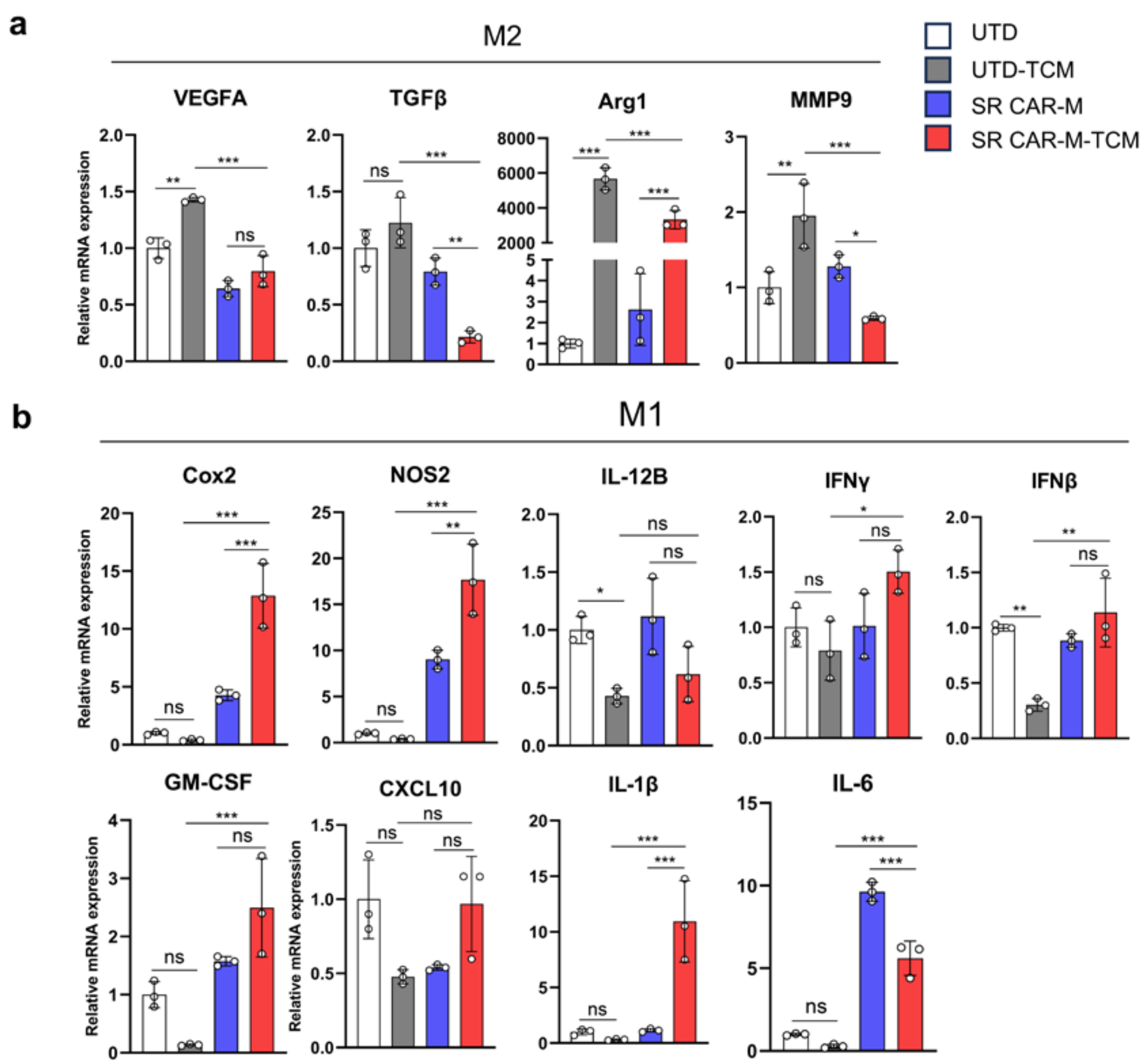

Supplementary Fig. 6: **SR CAR-M Can Switch to The M1 Phenotype Under Pressure from The Tumor Microenvironment.**

a-b. The mRNA expression of (**a**) M2 and M1(**b**) macrophage-related markers in UTD and SR CAR-M was detected by QPCR. All values are expressed as mean ± SEM from n = 3 independent biological replicates, with each biological replicate performed in technical triplicate. *P < 0.05, **P < 0.01, and ***P < 0.001.

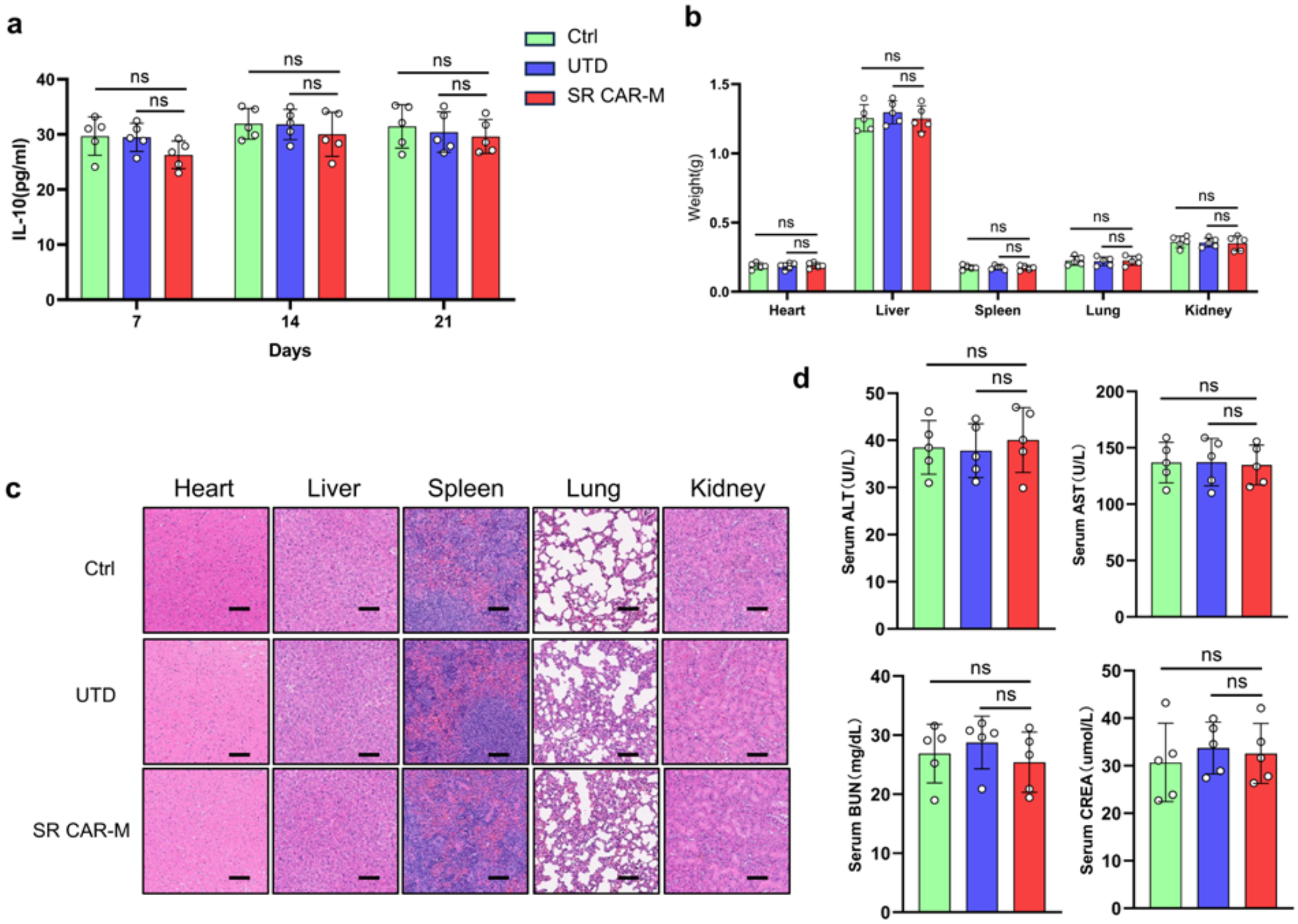

Supplementary Fig. 7: SR CAR-Ms maintain an antitumor phenotype within the TME

a. The level of IL-10 was checked by Elisa in mice that treated with SR CAR-M three times (7,14,21 days after tumor implant). **b**. Organ weights were recorded. **c**. Representative H&E images of normal organs from mice with indicated treatments. Scale bar=100 mm. **d**. Analysis of serum biomarkers for liver and kidney function in mice from the control, UTD and SR CAR-M groups. All values are expressed as mean ± SEM from n = 5 independent biological replicates, with each biological replicate performed in technical triplicate. *P < 0.05, **P < 0.01, and ***P < 0.001.

a

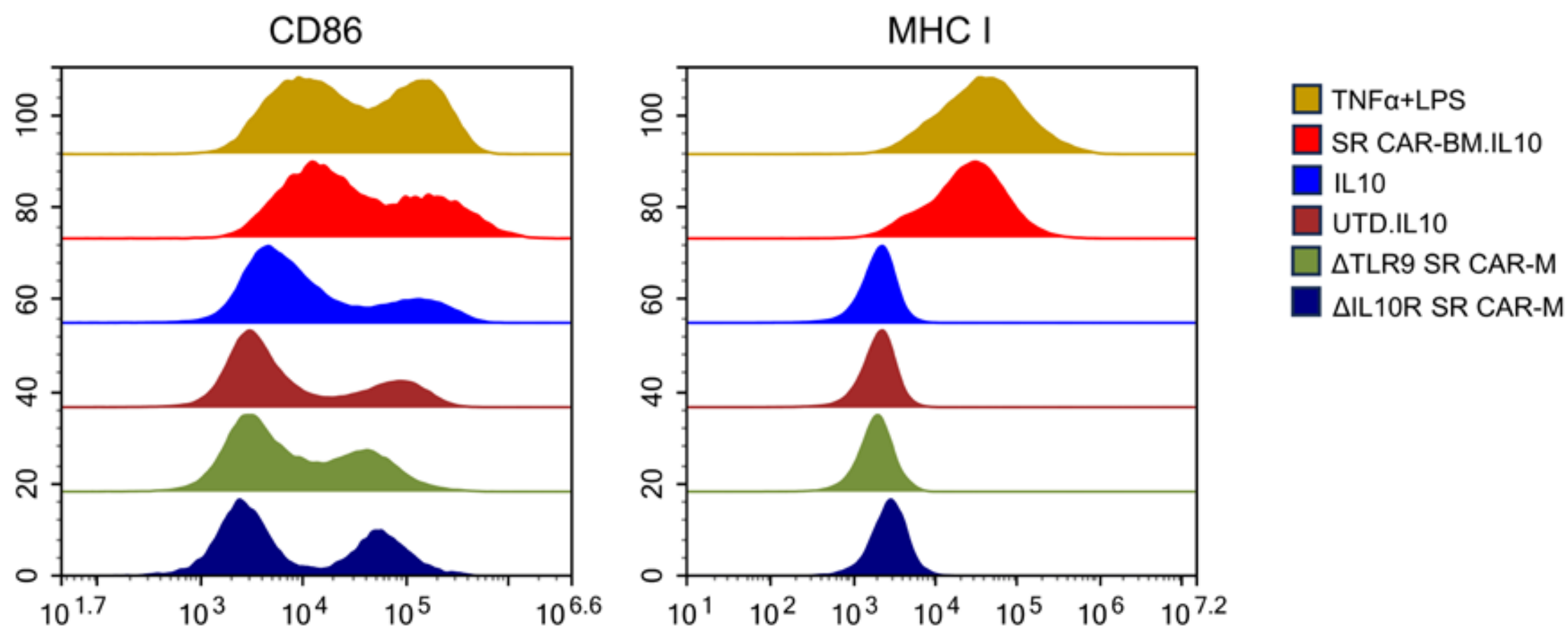

Supplementary Fig. 8: Maturation of bone marrow-derived dendritic cells induced by SR CAR-BM, ΔTLR9 SR CAR-M and ΔIL10R SR CAR-M

a. The levels of BMDC maturation markers (including MHC I and CD86) were detected using flow cytometry under different induction conditions.

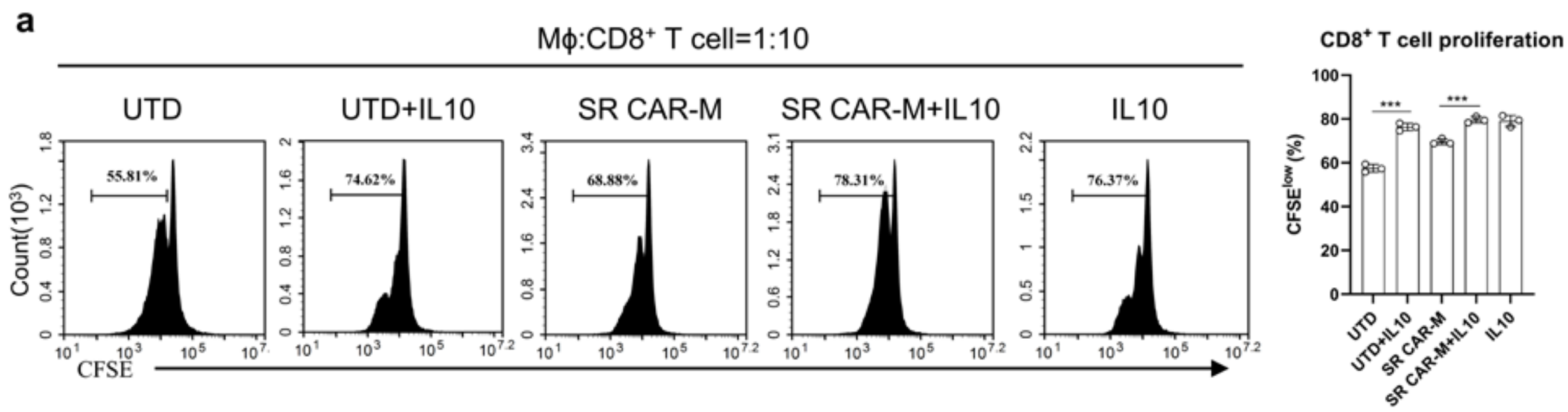

Supplementary Fig. 9: **IL-10 Promoted CD8+ T-Cell Proliferation.**

a. Proliferation of CD8+ T cells after co-culture with UTD or SR CAR-M in the presence or absence of IL-10 for three days. All values are expressed as mean ± SEM from n = 3 independent biological replicates, with each biological replicate performed in technical triplicate. *P < 0.05, **P < 0.01, and ***P < 0.001.

a

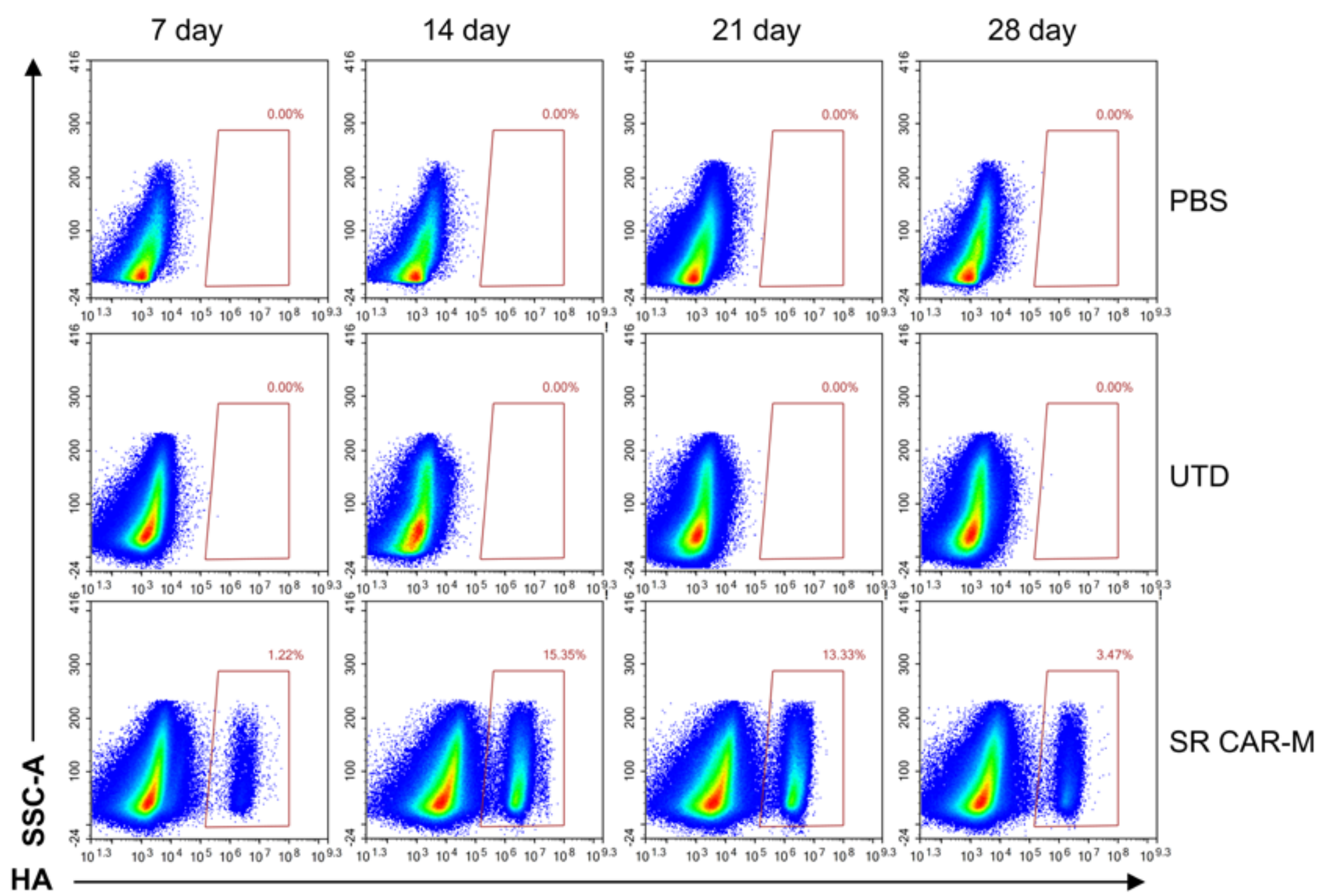

Supplementary Fig. 10: Evaluation of SR CAR-M infiltration

a. Representative flow cytometric analysis of SR CAR-M infiltration efficiency (HA-tag expression).

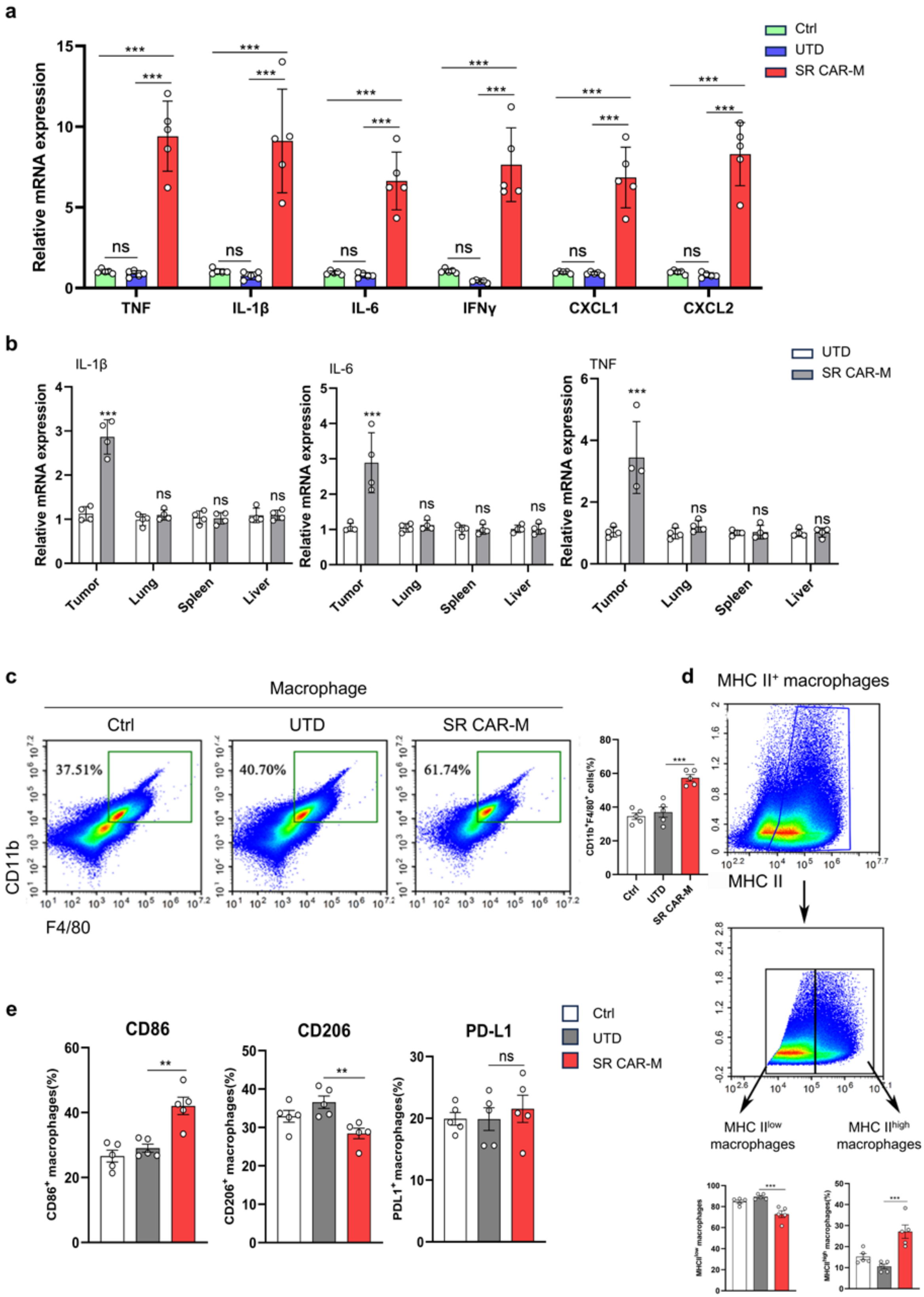

a
Ctrl
UTD
SR CAR-M
Relative mRNA expression
TNF
IL-1β
IL-6
IFNγ
CXCL1
CXCL2
b
UTD
SR CAR-M
IL-1β
IL-6
TNF
Relative mRNA expression
Tumor
Lung
Spleen
Liver
c
Macrophage
Ctrl
UTD
SR CAR-M
37.51%
40.70%
61.74%
CD11b
F4/80
CD11b+F4/80+ cells(%)
d
MHC II+ macrophages
MHC II
MHC IIlow macrophages
MHC IIhigh macrophages
e
CD86
CD206
PD-L1
CD86+ macrophages(%)
CD206+ macrophages(%)
PDL1+ macrophages(%)

Supplementary Fig. 11: SR CAR-M Regulates the Polarization of Tumor-Associated Macrophages in Tumor-Bearing Mice.

a. Quantification of M1 markers by qPCR in macrophages isolated from the tumor of mice in Ctrl, UTD and SR CAR-M groups. **b**. UTD and CAR-Ms were sorted from various organs 72 h after injection. The M1-like phenotype of macrophages from various organs was assessed using qPCR. **c**. Flow cytometry was used to detect the infiltration in the TME of 4T1 tumor-bearing mice treated with PBS, UTD, or SR CAR-Ms. **d**. Flow cytometry gating strategy for MHC high and low macrophages (up). The proportions of MHC high and low macrophages were detected and analyzed using flow cytometry (Down). **e**. The TAM phenotype was assessed by detecting the levels of phenotype-related markers (CD86, CD206, and PD-L1). All values are expressed as mean ± SEM from n = 5 independent biological replicates, with each biological replicate performed in technical triplicate (Except **b**, which is n=4). $^*P < 0.05$, $^{**}P < 0.01$, and $^{***}P < 0.001$.

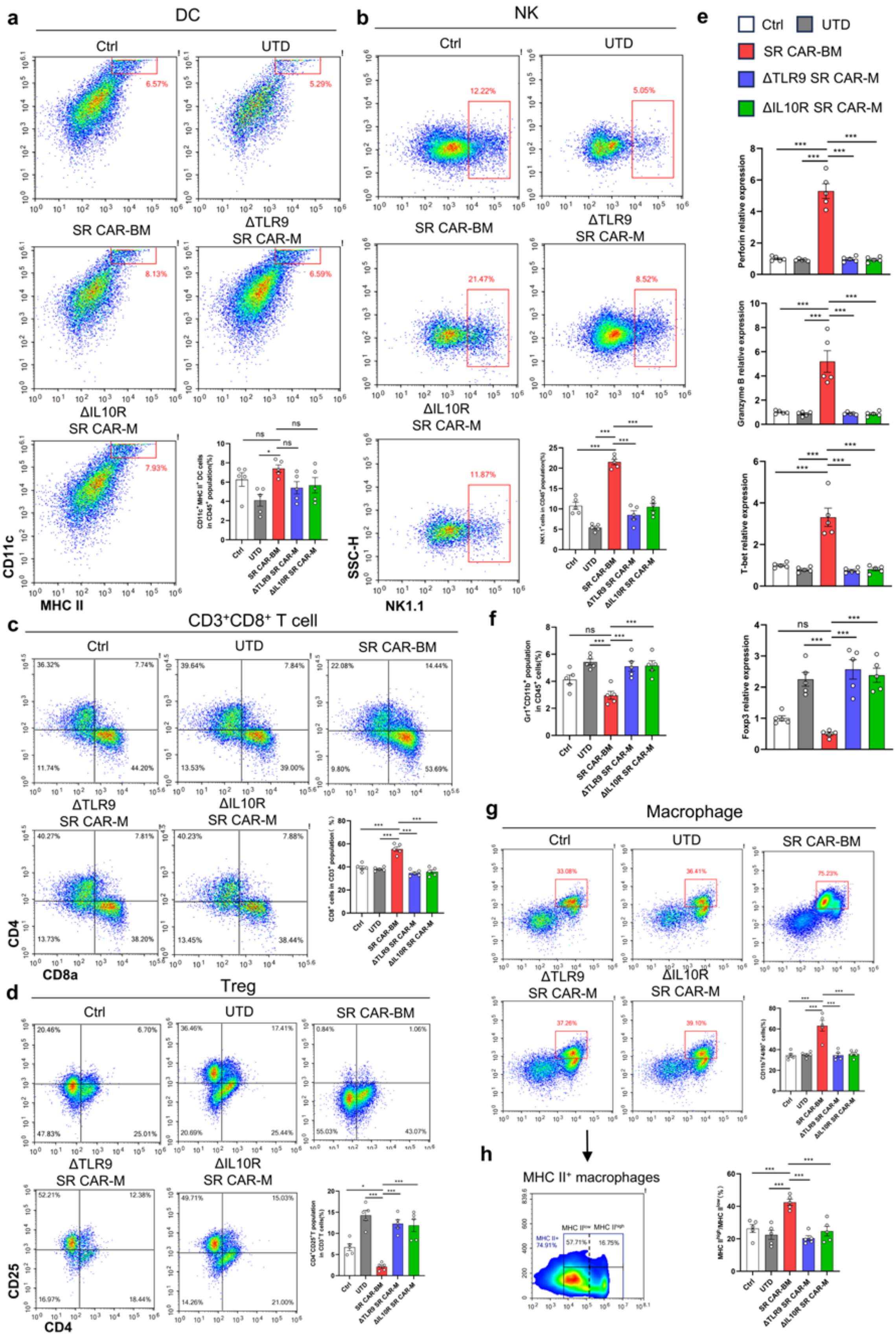

a
DC
Ctrl
UTD
6.57%
5.29%
SR CAR-BM
ΔTLR9 SR CAR-M
8.13%
6.59%
ΔIL10R SR CAR-M
7.93%
CD11c
MHC II
b
NK
Ctrl
UTD
12.22%
5.05%
SR CAR-BM
ΔTLR9 SR CAR-M
21.47%
8.52%
ΔIL10R SR CAR-M
11.87%
SSC-H
NK1.1
e
Ctrl
UTD
SR CAR-BM
ΔTLR9 SR CAR-M
ΔIL10R SR CAR-M
Perforin relative expression
Granzyme B relative expression
T-bet relative expression
Foxp3 relative expression
c
CD3⁺CD8⁺ T cell
Ctrl
UTD
SR CAR-BM
ΔTLR9 SR CAR-M
ΔIL10R SR CAR-M
CD4
CD8a
f
d
Treg
Ctrl
UTD
SR CAR-BM
ΔTLR9 SR CAR-M
ΔIL10R SR CAR-M
CD25
CD4
g
Macrophage
Ctrl
UTD
SR CAR-BM
ΔTLR9 SR CAR-M
ΔIL10R SR CAR-M
h
MHC II⁺ macrophages

Supplementary Fig.12: Evaluation of TME in tumor-bearing mice treated by SR CAR-BM, ΔTLR9 SR CAR-M and ΔIL10R SR CAR-M

a-d. Single-cell suspensions were prepared from tumors, and flow cytometry was used to detect the infiltration of DCs (**a**), NK cells (**b**), CD3+CD8+ T cells (**c**) and Tregs(**d**) into the TME of 4T1 tumor-bearing mice treated with PBS, SR CAR-BM, ΔTLR9 SR CAR-M, ΔIL10R SR CAR-M or UTD. **e**. The transcription levels of T-bet, Granzyme B, Perforin, and Foxp3 in tumor-bearing mice were detected by qPCR. **f-g**. Flow cytometry was used to detect the infiltration of (**f**) MDSCs and (**g**) TAMs into the TME of 4T1 tumor-bearing mice treated with PBS, SR CAR-BM, ΔTLR9 SR CAR-M, ΔIL10R SR CAR-M or UTD. **h**. The flow cytometry gate strategy for MHC high and low macrophages (left). The proportions of MHC II high/ MHC II low macrophages were detected and analyzed by flow cytometry (Right). All values are expressed as mean ± SEM from n = 5 independent biological replicates, with each biological replicate performed in technical triplicate. *P < 0.05, **P < 0.01, and ***P < 0.001.

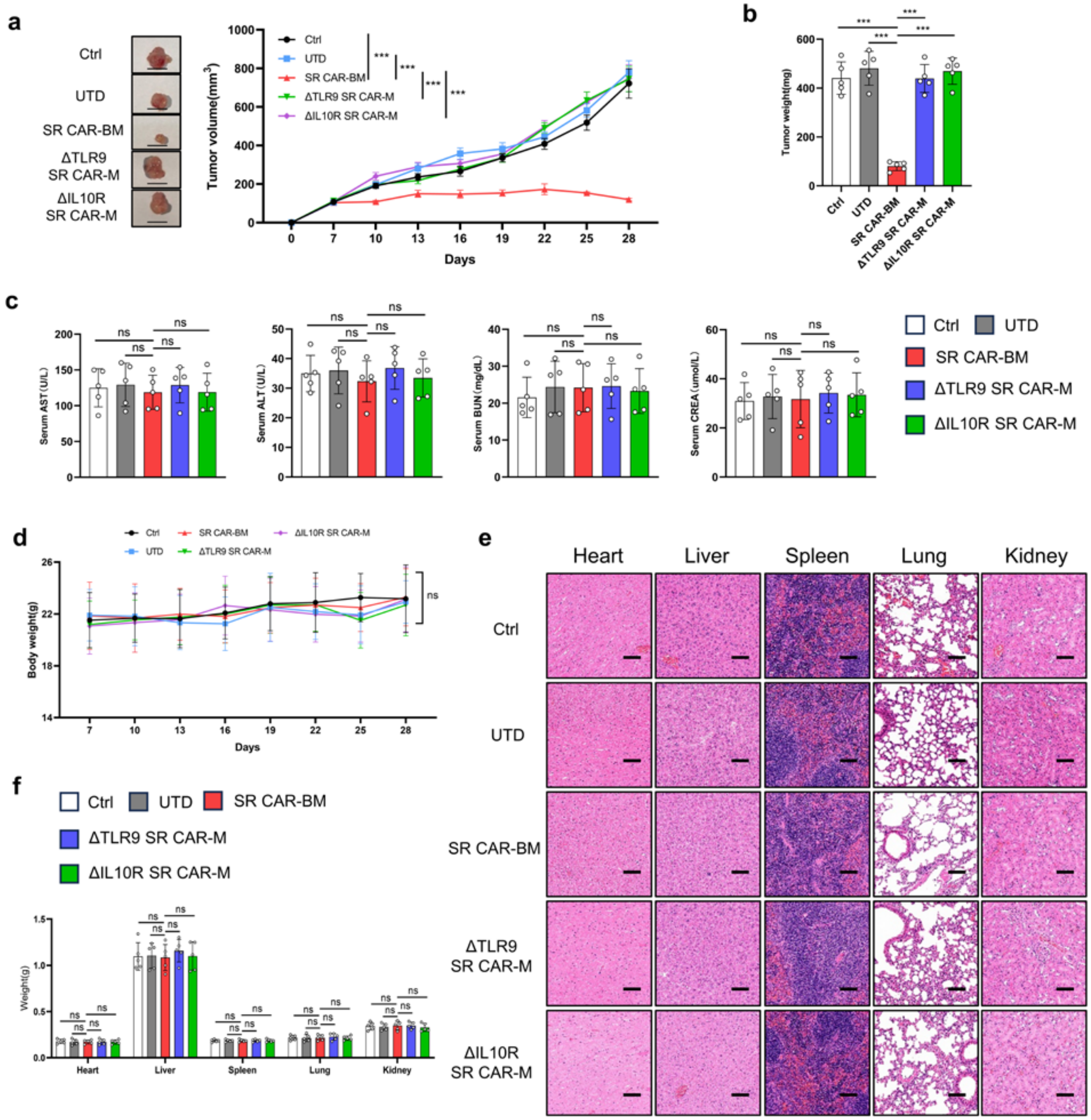

Supplementary Fig.13: Assessment of the anti-tumor capacity and safety of SR CAR-BM, ΔTLR9 SR CAR-M and ΔIL10R SR CAR-M

a. Representative photographs of tumor tissues isolated at the time of termination of the experiment (left; Scale bar=1 cm) (**a**, left). Mean tumor volume was recorded at the indicated times (**a**, right). **b**. Tumor weight of mice in PBS, SR CAR-BM, ΔTLR9 SR CAR-M, ΔIL10R SR CAR-M or UTD groups was measured. **c**. Analysis of serum biomarkers for liver and kidney function in mice from PBS, SR CAR-BM, ΔTLR9 SR CAR-M, ΔIL10R SR CAR-M or UTD groups. **d**. Body weights

of mice from each group. **e-f**. Representative H&E images of normal organs from mice with indicated treatments (**e**), and organ weights were recorded (**f**). Scale bar=100 mm. All values are expressed as mean ± SEM from n = 5 independent biological replicates, with each biological replicate performed in technical triplicate. *P < 0.05, **P < 0.01, and ***P < 0.001.

Supplementary Table 1. Murine Q-PCR primers

Gene	Direction	Sequence (5'→3')
IL10RA	Forward	CCAAACCAGTCTGAGAGCACCT
IL10RA	Reverse	CAGGACAATGCCTGAGCCTTTC
IL10RB	Forward	CTGTGAACGGACAGGCAATGAC
IL10RB	Reverse	ATGAGCCACAGCACGACAAAGC
SOCS3	Forward	GGACCAAGAACCTACGCATCCA
SOCS3	Reverse	CACCAGCTTGAGTACACAGTCG
PD-L1 (CD274)	Forward	TGCGGACTACAAGCGAATCACG
PD-L1 (CD274)	Reverse	CTCAGCTTCTGGATAACCCTCG
COX2 (PTGS2)	Forward	GCGACATACTCAAGCAGGAGCA
COX2 (PTGS2)	Reverse	AGTGGTAACCGCTCAGGTGTTG
NOS2	Forward	GAGACAGGGAAGTCTGAAGCAC
NOS2	Reverse	CCAGCAGTAGTTGCTCCTCTTC
IL-1β	Forward	TGGACCTTCCAGGATGAGGACA
IL-1β	Reverse	GTTCATCTCGGAGCCTGTAGTG
IL-6	Forward	TACCACTTCACAAGTCGGAGGC
IL-6	Reverse	CTGCAAGTGCATCATCGTTGTTC
GM-CSF (CSF2)	Forward	AACCTCCTGGATGACATGCCTG
GM-CSF (CSF2)	Reverse	AAATTGCCCCGTAGACCCTGCT
CD80	Forward	CCTCAAGTTTCCATGTCCAAGGC
CD80	Reverse	GAGGAGAGTTGTAACGGCAAGG
CD86	Forward	ACGTATTGGAAGGAGATTACAGCT
CD86	Reverse	TCTGTCAGCGTTACTATCCCGC
TNF	Forward	GGTGCCTATGTCTCAGCCTCTT
TNF	Reverse	GCCATAGAACTGATGAGAGGGAG
IL-12β	Forward	TTGAACTGGCGTTGGAAGCACG
IL-12β	Reverse	CCACCTGTGAGTTCTTCAAAGGC
IFN-γ	Forward	CAGCAACAGCAAGGCGAAAAAGG
IFN-γ	Reverse	TTTCCGCTTCCTGAGGCTGGAT
IFN-β	Forward	GCCTTTGCCATCCAAGAGATGC
IFN-β	Reverse	ACACTGTCTGCTGGTGGAGTTC
CCL2	Forward	GCTACAAGAGGATCACCAGCAG
CCL2	Reverse	GTCTGGACCCATTCCTTCTTGG
CXCL1	Forward	TCCAGAGCTTGAAGGTGTTGCC
CXCL1	Reverse	AACCAAGGGAGCTTCAGGGTCA
CXCL2	Forward	CATCCAGAGCTTGAGTGTGACG
CXCL2	Reverse	GGCTTCAGGGTCAAGGCAAACT

CXCL10	Forward	ATCATCCCTGCGAGCCTATCCT
CXCL10	Reverse	GACCTTTTTTGGCTAAACGCTTTC
Arg1	Forward	CATTGGCTTGCGAGACGTAGAC
Arg1	Reverse	GCTGAAGGTCTCTTCCATCACC
VEGFA	Forward	CTGCTGTAACGATGAAGCCCTG
VEGFA	Reverse	GCTGTAGGAAGCTCATCTCTCC
TGF-β	Forward	TGATACGCCTGAGTGGCTGTCT
TGF-β	Reverse	CACAAGAGCAGTGAGCGCTGAA
CD163	Forward	GGCTAGACGAAGTCATCTGCAC
CD163	Reverse	CTTCGTTGGTCAGCCTCAGAGA
CD206 (MRC1)	Forward	GTTCACCTGGAGTGATGGTTCTC
CD206 (MRC1)	Reverse	AGGACATGCCAGGGTCACCTTT
MMP9	Forward	GCTGACTACGATAAGGACGGCA
MMP9	Reverse	TAGTGGTGCAGGCAGAGTAGGA
IL-10	Forward	CGGGAAGACAATAACTGCACCC
IL-10	Reverse	CGGTTAGCAGTATGTTGTCCAGC
IL-4	Forward	ATCATCGGCATTTTGAACGAGGTC
IL-4	Reverse	ACCTTGGAAGCCCTACAGACGA
CD69	Forward	GGGCTGTGTTAATAGTGGTCCTC
CD69	Reverse	CTTGCAGGTAGCAACATGGTGG
Granzyme B	Forward	CAGGAGAAGACCCAGCAAGTCA
Granzyme B	Reverse	CTCACAGCTCTAGTCCTCTTGG
Perforin	Forward	ACACAGTAGAGTGTCGCATGTAC
Perforin	Reverse	GTGGAGCTGTTAAAGTTGCGGG
T-bet (TBX21)	Forward	CCACCTGTTGTGGTCCAAGTTC
T-bet (TBX21)	Reverse	CCACAAACATCCTGTAATGGCTTG
Foxp3	Forward	CCTGGTTGTGAGAAGGTCTTCG
Foxp3	Reverse	TGCTCCAGAGACTGCACCACTT
PD-1 (PDCD1)	Forward	CGGTTTCAAGGCATGGTCATTGG
PD-1 (PDCD1)	Reverse	TCAGAGTGTCGTCCTTGCTTCC
LAG-3	Forward	CTCCATCACGTACAACCTCAAGG
LAG-3	Reverse	GGAGTCCACTTGGCAATGAGCA
Tim-3 (HAVCR2)	Forward	ACAGACACTGGTGACCCTCCAT
Tim-3 (HAVCR2)	Reverse	CAGCAGAGACTCCCACTCCAAT
TIGIT	Forward	CCACAGCAGGCACGATAGATA
TIGIT	Reverse	CATGCCACCCCAGGTCAAC
CTLA-4	Forward	GTACCTCTGCAAGGTGGAACTC
CTLA-4	Reverse	CCAAAGGAGGAAGTCAGAATCCG
β-Actin	Forward	GTGACGTTGACATCCGTAAAGA

β-Actin	Reverse	GCCGGACTCATCGTACTCC

Supplementary Table 2. Human Q-PCR primers

Gene	Direction	Sequence (5'→3')
IL10RA	Forward	CCTCCGTCTGTGTGGTTTGAA
IL10RA	Reverse	CACTGCGGTAAGGTCATAGGA
IL10RB	Forward	TACCACCTCCCGAAAATGTCA
IL10RB	Reverse	CCCAGTCTGAATGCTCATCTG
SOCS3	Forward	CCTGCGCCTCAAGACCTTC
SOCS3	Reverse	GTCACTGCGCTCCAGTAGAA
PD-L1 (CD274)	Forward	TGCCGACTACAAGCGAATTACTG
PD-L1 (CD274)	Reverse	CTGCTTGTCCAGATGACTTCGG
COX2 (PTGS2)	Forward	CGGTGAAACTCTGGCTAGACAG
COX2 (PTGS2)	Reverse	GCAAACCGTAGATGCTCAGGGA
IL-1β	Forward	CCACAGACCTTCCAGGAGAATG
IL-1β	Reverse	GTGCAGTTCAGTGATCGTACAGG
IL-6	Forward	AGACAGCCACTCACCTCTTCAG
IL-6	Reverse	TTCTGCCAGTGCCTCTTTGCTG
GM-CSF (CSF2)	Forward	GGAGCATGTGAATGCCATCCAG
GM-CSF (CSF2)	Reverse	CTGGAGGTCAAACATTTCTGAGAT
CD80	Forward	AAACTCGCATCTACTGGCAAA
CD80	Reverse	GGTTCTTGTACTCGGGCCATA
Arg1	Forward	TCATCTGGGTGGATGCTCACAC
Arg1	Reverse	GAGAATCCTGGCACATCGGGAA
VEGFA	Forward	TTGCCTTGCTGCTCTACCTCCA
VEGFA	Reverse	GATGGCAGTAGCTGCGCTGATA
TGF-β	Forward	TACCTGAACCCGTGTTGCTCTC
TGF-β	Reverse	GTTGCTGAGGTATCGCCAGGAA
GAPDH	Forward	GTCTCCTCTGACTTCAACAGCG
GAPDH	Reverse	ACCACCCTGTTGCTGTAGCCAA

Supplementary Table 3. The sequence of siRNA for Myd88 knockdown

**Gene**	**Direction**	**Sequence (5'→3')**
siRNA-1	Forward	GCGACUGAUUCCUAUUAAAdTdT
siRNA-1	Reverse	UUUAAUAGGAAUCAGUCGCdTdT
siRNA-2	Forward	GGAGCUGAAGUCGCGCAUCdTdT
siRNA-2	Reverse	GAUGCGCGACUUCAGCUCCdTdT
siRNA-3	Forward	CAGAAGCGACUGAUUCCUAdTdT
siRNA-3	Reverse	UAGGAAUCAGUCGCUUCUGdTdT
